# Greenhouse gas emissions in relation to micronutrient intake and implications of energy intake: a comparative analysis of different modeling approaches

**DOI:** 10.1016/j.ajcnut.2025.02.031

**Published:** 2025-03-10

**Authors:** Anna Stubbendorff, Elinor Hallström, Georgia Tomova, Yan Borné, Suzanne Janzi, Emily Sonestedt, Ulrika Ericson

**Affiliations:** 1 Nutritional Epidemiology, Department of Clinical Sciences Malmö, Lund University, Malmö, Sweden; 2 Nutrition, Sustainability and Health Promotion Group, National Food Institute, Technical University of Denmark, Kgs Lyngby, Denmark; 3 Department of Agriculture and Food, Research Institutes of Sweden (RISE), Lund, Sweden; 4 Leeds Institute for Data Analytics, University of Leeds, Leeds, UK; 5 School of Food Science & Nutrition, University of Leeds, Leeds, UK; 6 Department of Food and Meal Science, Faculty of Natural Science, Kristianstad University, Kristianstad, Sweden; 7 Diabetes and Cardiovascular disease, Department of Clinical Sciences Malmö, Lund University, Malmö, Sweden

**Keywords:** sustainable diet, climate, micronutrient deficiency, nutrient adequacy, nutrient intake, energy intake

## Abstract

**Background:**

Human diets account for 30% of greenhouse gas emissions (GHGE). Reporting dietary GHGE with or without energy standardization yields different outcomes, often resulting in conflicting conclusions regarding associations with micronutrient intake.

**Objectives:**

This study aims to compare methods of reporting dietary GHGE, with and without consideration of energy intake, and their respective associations with micronutrient intake.

**Methods:**

Data were sourced from the Malmö Diet and Cancer Study, a cohort involving 25,970 participants. GHGE were estimated based on life cycle assessment data. The study explores different methods of reporting dietary climate impact: GHGE per day, GHGE per 1000 kcal, and with different energy adjustments. Association with micronutrient intake was modeled as daily intake and per 1000 kcal using linear regression models.

**Results:**

Diets with higher GHGE per day were associated with a higher daily intake of all 17 examined micronutrients. When energy was included in the model, the results for GHGE per 1000 kcal aligned well with those for GHGE per day. However, using GHGE per 1000 kcal generally showed that higher GHGE were linked to lower daily micronutrient intake. Different methods of adjusting for energy intake yielded estimates with varying directions and magnitudes of associations.

**Conclusions:**

This study highlights the implications of energy intake when assessing the impact of dietary GHGE and demonstrates that the choice of GHGE modeling approach might have important consequences for the results and interpretation. The method of choice for modeling dietary GHGE in relation to micronutrient intake needs to be carefully considered in future studies.

## Introduction

Climate change increasingly threatens sustainability and human health. Food systems contribute to climate change, accounting for approximately one-third of global anthropogenic greenhouse gas emissions (GHGE) [1,2]. An increased proportion of plant-based foods could substantially reduce dietary GHGE and increase chances to keep environmental systems within the planetary boundaries [3,4]. However, concern has been raised that a dietary shift toward more climate-friendly diets, low in animal-sourced foods, might threaten nutritional adequacy and increase risk of nutrient deficiencies, because some animal-based foods might contain micronutrients that cannot be sourced in adequate amounts from plant-based foods [5]. Excess food demand resulting from overconsumption or food waste contributes to increased dietary GHGE. It has been suggested that obesity contributes to ∼20% more dietary GHGE compared with a normal weight state [6] and that overconsumption linked to overweight and obesity accounts for 1.6% of total global GHGE.

**FIGURE 1:**
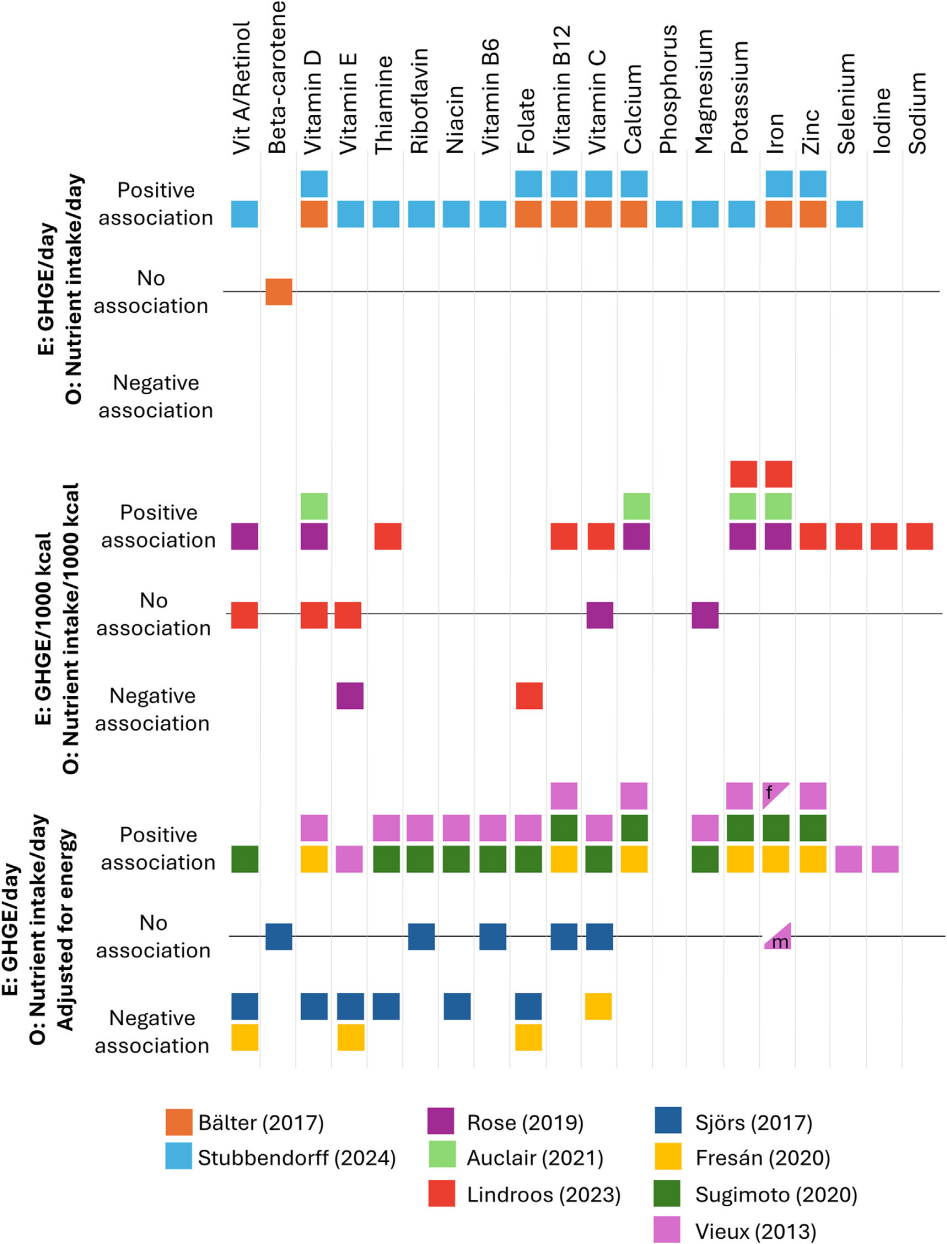
Examples of approaches for reporting dietary greenhouse gas emissions (GHGE) in previous observational studies, in relation to micronutrient intake [[8], [9], [10], [11], [12], [13], [14], [15], [16]]. E, exposure; f, female; m, male; O, outcome.

Four major approaches to assessing the sustainability of diets have been previously suggested: analyzing the characteristics of hypothetical diets, evaluating the characteristics of existing diets, identifying existing "positive deviants," and designing diets through optimization [7]. Within the approach of studying existing diets, different methodologies can be employed to model dietary GHGE in relation to micronutrient intake. Estimates from these different approaches might vary considerably, leading to ostensibly different findings and potentially conflicting conclusions regarding the environmental and nutritional implications of diets (Figure 1) [[8], [9], [10], [11], [12], [13], [14], [15], [16]]. One method of reporting dietary climate impact is to calculate the daily dietary GHGE, by summarizing GHGE from different foods eaten and reporting the average daily emissions. This approach provides an estimate of the total environmental impact based on the quantity and type of food consumed. Using this method, it has been shown that higher GHGE are associated with a higher micronutrient intake [8,9]. However, this could be driven by the fact that simply eating more, on average, would lead to both higher GHGE and higher micronutrient intake. This is a common challenge in nutritional epidemiology, and different energy adjustment methods are often used to enable comparisons within the same or similar levels of energy intake [17]. Another approach is therefore to report dietary GHGE per 1000 kcal of food consumed, which accounts for the environmental impact relative to the energy content of the diet [10,11]. This approach rescales the exposure as a proportion of total energy and does not take the total food consumption into account.

Finally, adjusting for dietary energy intake in the statistical model directly (as opposed to dividing by it) has also been used when reporting GHGE from diets. The primary reason for adjusting for total energy intake is to control for unobserved confounding factors that may be best approximated by the total caloric intake [18]. Energy adjustment might overcome the problem of unobserved confounding, but it brings additional interpretational challenges and might be less straightforward. Studies that report energy-adjusted estimates also tend to show conflicting results. In one study from Japan using energy adjustment, higher GHGE were associated with increased nutrient intake [12]. Contradictory, participants with lower-GHGE diets tended to have slightly more nutritious diets in Sweden [13] and in France [14]. In another study with energy adjustment, lower-GHGE were associated with lower intakes of some vitamins and all minerals, but higher levels of others [15]. In addition to variations in how the exposure is modeled, studies also differ in how they report nutrient intake. Nutrient intake may be presented as daily intake, as intake per 1000 kcal, or as the proportion of participants exceeding the average requirement (AR) or recommended intake (RI).

Given the divergent methodologies and the varying results they produce, there is a critical need to systematically compare these approaches to better understand their implications. This discrepancy underscores the need for a comprehensive understanding of how different approaches to accounting for energy intake may impact the apparent relationship between dietary climate impact and micronutrient intake. This study aims to fill this gap by exploring the different methods of reporting dietary climate impact (GHGE per day, GHGE per 1000 kcal, and GHGE per day, adjusting for total energy in different ways) and examining the association with micronutrient intake (as daily intake and per 1000 kcal) that results from each of the modeling approaches. The overall aim is to provide an advanced understanding of the nutritional impacts of climate-friendly diets. By elucidating the strengths and limitations of each approach, this research seeks to inform the development of more effective and comprehensive sustainability assessments of dietary patterns.

## Methods

### Malmö Diet and Cancer Study

The Malmö Diet and Cancer (MDC) Study is a prospective cohort study with recruitment and baseline examinations between 1991 and 1996. The source population included 74,138 individuals, entire birth cohorts of females born between 1923 and 1950 (age 45–73) and males born between 1923 and 1945 (age 46–73) living in Malmö at the time. Individuals with limited Swedish proficiency or mental disabilities, which prevented them from completing the baseline questionnaire, were excluded, leaving 68,905 eligible individuals. Detailed descriptions of the recruitment process for the MDC have been published elsewhere [19,20]. Ultimately, 28,098 subjects completed the baseline examinations, representing 41% of the eligible individuals. For this specific study, participants with incomplete food consumption information (*n* = 2128) were further excluded, resulting in a final study population of 25,970 individuals, of which 61% are female (Supplemental Figure 1). The participants provided written informed consent, and ethical approval for the study was obtained (LU 51–90).

### Baseline examinations

At baseline, participants completed a self-administered questionnaire to provide information on their demographic, lifestyle, socioeconomic, and social factors, as well as their current health and medical history. Instructions were provided on how to complete a food diary and a food frequency questionnaire (FFQ). Anthropometric measurements, blood pressure, and blood samples were collected during the first visit [21]. Approximately 2 wk later, during the second visit, a dietary interview was conducted, and the questionnaires were reviewed [19,21].

### Dietary assessment and analysis of nutrient intake

Dietary intake was evaluated during baseline examinations using a validated, modified diet history [20]. The method comprises 3 components: *1*) a 7-d food diary to record intakes of prepared meals such as lunch and dinner, cold beverages (including alcoholic beverages), and dietary supplements; *2*) a 168-item FFQ assessing consumption frequencies and portion sizes of foods not included in the food diary such as breakfast and snacks, over the last year; *3*) a 45- or 60-min interview depending on the year of assessment, to further assess portion sizes, food choices, and cooking methods. Each participant’s FFQ was checked for missing values and cross-verified with the food diary to avoid counting the same foods twice. The recorded food intakes from both the food diary and FFQ were summarized, and the average intake of individual foods was expressed in grams per day. Energy and nutrient intake calculations were performed using a food composition database comprising 1600 food items (PC-KOST2-93) from the Swedish Food Agency. Extreme values in the reporting of major food groups, portion sizes, total energy, and nutrients were checked for errors and corrected. Protein, fat, and carbohydrate amounts were converted to percentages of nonalcoholic energy intake (E%). A similar modified diet history method used in the MDC has previously shown good ranking validity and reproducibility when compared with an 18-d weighed food records reference method [22,23]. Compared with the reference method, the MDC method yielded an 18% higher absolute energy intake at the group level [23]. Reported food intakes were generally higher than those recorded by the reference method, except for fish, cream, and alcohol, as well as meat in females, and rice, pasta, and eggs in males [23].

In September 1994, the coding routines for dietary data were adjusted, reducing the dietary interview from 60 to 45 min. Thus, a variable indicating the assessment version (old or new) was introduced. Although the assessed energy intake was slightly lower after this change, it did not affect participant rankings [24]. A variable with 4 categories representing the season at baseline assessments was created to control for seasonal variations.

### Climate impact assessment

The dietary climate impact was estimated using life cycle assessment (LCA) data expressing the GHGE in kilograms of carbon dioxide equivalents per kilogram of food (kg CO_2_eq/kg). The LCA data were compiled and harmonized as described by Hallström et al. [25], and selected to represent the average Swedish consumption, considering current production methods and origins. The system boundaries encompassed GHGE from cradle to consumer, including emissions from primary production, energy use in processing, packaging, international transportation to Sweden, national transportation and distribution, and home transportation by the consumer, including food losses and wastage throughout the food system. Emissions from home cooking were excluded because most food intake was measured in raw weight. For certain food items, additional LCA data from other sources were incorporated and harmonized to align with respect to functional unit and system boundaries [26,27]. Reported food intake was matched with LCA data by adjusting for weight changes during cooking. For grouped food items (for example, rice and pasta, fresh fruits), consumption proportions were estimated based on a Swedish dietary survey from 1997 to 1998 [28]. The climate impact of spices, stock, and vinegar was excluded because of limited data. Climate impact calculations covered 117 subgroups of food items, using dietary intake data from the MDC. GHGE from dietary supplements were not included. To compare GHGE across different food groups, all foods were categorized into 12 groups (Supplemental Table 1), which were further merged into 3 main categories to compare the impacts of plant-based, animal-based, and discretionary foods.

### Statistical analyses

Dietary climate impact was reported as GHGE per day, as a continuous variable. To account for energy intake, we applied multiple approaches. First, we included total energy intake as a covariate in the statistical model. Second, we used the residual method to create an energy-adjusted GHGE variable, which was analyzed both with and without total energy intake as a covariate. Finally, we calculated GHGE per 1000 kcal and modeled it with and without total energy intake as a covariate.

To limit the influence of extreme values, the participants were further divided into quintiles based on their daily dietary GHGE, and another set of quintiles was created based on the GHGE per 1000 kcal (referred to as Q1–Q5). Quintiles were gender specific because males and females are known to emit different levels of GHGE due to differences in energy intake, dietary habits, and micronutrient recommendations. For quintiles of GHGE per day and per 1000 kcal, estimated marginal means were reported. Individual dietary micronutrient intake was compared with gender- and age-specific (51–70 y) reference values for AR and RI as outlined in the Nordic Nutrition Recommendations from 2023 [29] using logistic regression.

In all the aforementioned analyses, dietary micronutrient intake per day and micronutrient intake per 1000 kcal were explored as outcomes. Micronutrient intake was reported as intake of micronutrients from dietary sources, excluding micronutrient supplements. Micronutrient intakes were analyzed using linear regression, and β-coefficients were reported for the different models. We included age (years), season [4 categories: winter (December–February), spring (March–May), summer (June–August), and fall (September–November)], and dietary assessment version (old and new) as covariates in all analyses. All analyses were performed separately for males and females. Finally, Pearson’s correlation coefficients were reported between GHGE, energy intake, and micronutrient intake.

Statistical analyses were performed using Stata SE18 (StataCorp LLC). When *P* values are presented, a 2-sided *P* value lower than 0.05 was considered statistically significant.

### Sensitivity analyses

In sensitivity analyses, we modeled the quintiles of daily dietary GHGE and quintiles of GHGE per 1000 kcal as continuous variables and consequently reported the β-coefficients. As another sensitivity analysis, we excluded potential misreporters. Potential under- and over-reporters of energy intake were identified based on the method by Black and Goldberg [29], which uses individual physical activity level values. Specifically, participants were considered under- or over-reporters if their ratio of reported energy intake to basal metabolic rate fell outside the 95% confidence interval of their calculated physical activity level. A more detailed description of this method is available in a previous publication [30].

## Results

### Baseline characteristics and climate impact

**FIGURE 2:**
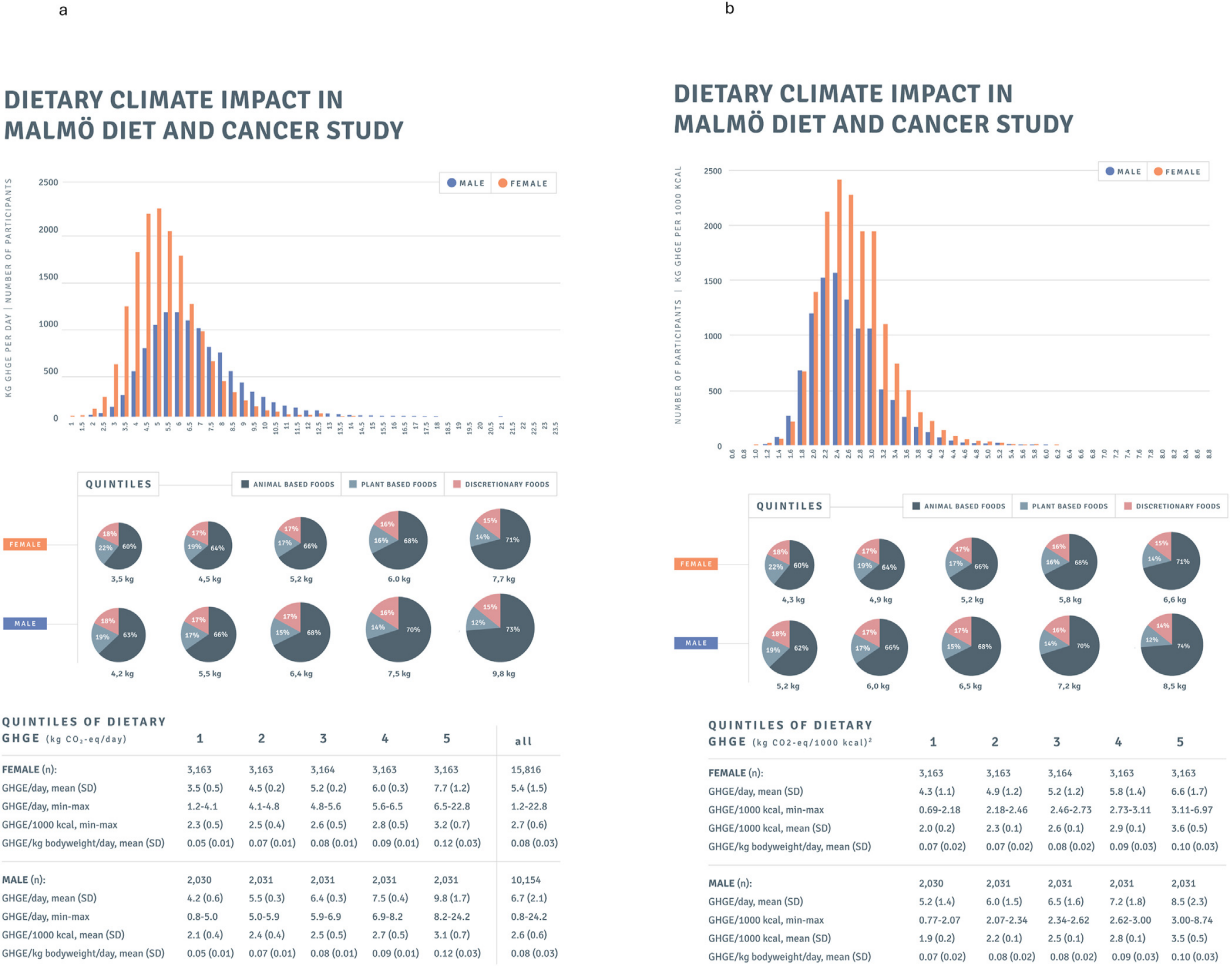
(A) Dietary climate impact according to quintiles in 25,970 participants from the Malmö Diet and Cancer Study. Proportion of dietary greenhouse gases (GHGE) from plant-based foods, animal-based foods, and discretionary foods,^1^ with the size of pies reflecting GHGE/d. The first quintile for females is used as the reference for the pie charts. ^1^Foods in animal-based group are red meat, poultry and egg, seafood, and dairy. In plant-based foods, vegetables, fruits, and berries and nuts, cereals, and added fats. In discretionary food, nonalcoholic drinks, alcoholic drinks, sweets and snacks, and other foods. (B) Dietary climate impact according to quintiles in 25,970 participants from the Malmö Diet and Cancer Study. Proportion of dietary greenhouse gases (GHGE) from plant-based foods, animal-based foods, and discretionary foods,^1^ with the size of pies reflecting GHGE/d. The first quintile for females is used as the reference for the pie charts. ^1^Foods in animal-based group are red meat, poultry and egg, seafood, and dairy. In plant-based foods, vegetables, fruits and berries and nuts, cereals, and added fats. In discretionary food, nonalcoholic drinks, alcoholic drinks, sweets and snacks, and other foods.

In this study, the mean age was 58 y in females and 59 y in males. The reported daily average energy intake was 2031 kcal for females and 2635 kcal for males, and the mean BMI was 25.4 kg/m^2^ in females and 26.3 kg/m^2^ in males. The mean dietary GHGE in all participants of MDC was 5.9 kg of CO_2_eq per participant per day, with females having a lower mean dietary GHGE of 5.4 kg, compared with males where the mean GHGE was 6.7 kg CO_2_eq per day (Figure 2A and B). However, expressed as GHGE per 1000 kcal, females had similar energy-standardized emissions as males (2.7 kg compared with 2.6 kg CO_2_eq/1000 kcal).

When comparing quintiles based on dietary GHGE per day, participants with higher dietary GHGE were more likely to be younger, have a university degree, and be smokers (Table 1). Higher quintiles were associated with higher energy intake and higher alcohol consumption. The proportion of total and saturated fat and protein (E%) was slightly higher in the highest quintiles of dietary GHGE, whereas the contribution from carbohydrates was lower. Measured in grams per day, the intake of protein was higher in diets with higher GHGE (Q5/Q1: females 96 g/58 g, males 122 g/72 g). For fiber, the total intake was higher in the highest quintiles of GHGE, whereas fiber/1000 kcal was lower. The mean contribution to GHGE from animal-based products was 71%/60% for females and 73%/63% for males in Q5/Q1 (Figure 2A, Supplemental Figure 2A). The mean GHGE per day in Q5 was 120% higher for females and 133% for males compared with Q1 (Figure 2A). Food consumption from all food groups was higher in Q5 than Q1 (Table 2). The consumption of red meat was 2-fold in Q5 compared with Q1 in both females and males.

**TABLE 1:** Participant characteristics according to quintiles of dietary greenhouse gas emissions (GHGE) per day in 25,970 participants from the Malmö Diet and Cancer Study.

	Quintiles of dietary climate impact (kg CO_2_eq^1^/d)^2^				
	1	2	3	4	5
Female (*n*)	3163	3163	3164	3163	3163
Age (y)	59.9 (8.1)	58.6 (8.2)	57.5 (7.9)	56.3 (7.7)	54.9 (7.0)
BMI^3^	25.8 (4.4)	25.6 (4.3)	25.5 (4.2)	25.1 (4)	25.2 (4.3)
BMI >25^3^ (%)	50.7	47.8	47.4	44.1	45.0
Current smokers^4^ (%)	25.4	26.3	27.7	28.5	33.1
High alcohol consumption^5^ (%)	0.4	1.0	1.8	2.4	6.4
High physical activity^6^ (%)	19.1	17.8	20.6	21.6	22.8
University degree^7^ (%)	9.4	12.4	14.4	17.6	21.6
Energy intake (kcal/d)	1578 (321)	1851 (322)	2013 (347)	2206 (393)	2506 (529)
Bodyweight (kcal/kg)	24.1 (6.7)	28.1 (7.1)	30.3 (7.3)	33.4 (8.2)	37.7 (10.8)
Fat E%^8^	36.2 (6.2)	37.1 (6)	37.8 (5.8)	38.5 (5.8)	39.4 (5.9)
Saturated fat E%^8^	15.3 (3.6)	16.0 (3.7)	16.4 (3.6)	16.9 (3.8)	17.5 (3.9)
Unsaturated fat E%^8^	18.6 (3.4)	18.7 (3.2)	19.0 (3.1)	19.1 (3)	19.4 (3)
Protein E%^8^	15.2 (2.6)	15.4 (2.4)	15.7 (2.4)	15.7 (2.4)	16.2 (2.5)
Carbohydrate E%^8^	48.6 (6.3)	47.5 (6)	46.5 (5.7)	45.8 (5.7)	44.5 (5.8)
Dietary fiber (g)	16.2 (5.9)	17.8 (5.4)	18.6 (5.5)	20.1 (6.1)	21.6 (6.8)
Dietary fiber (g/1000 kcal)	10.5 (3.2)	9.9 (2.8)	9.6 (2.5)	9.5 (2.5)	9.0 (2.5)
Male (*n*)	2030	2031	2031	2031	2031
Age (y)	62.4 (6.9)	60.7 (7.1)	59.4 (7)	58.1 (6.7)	56.4 (6.3)
BMI^3^	26.3 (3.5)	26.2 (3.4)	26.2 (3.5)	26.2 (3.4)	26.4 (3.7)
BMI >25^3^ (%)	63.6	62.0	63.1	61.3	62.0
Current smokers^4^ (%)	23.3	26.7	27.0	31.0	35.6
High alcohol consumption^5^ (%)	1.6	3.6	6.6	8.9	16.5
High physical activity^6^ (%)	20.6	21.3	20.1	19.9	20.5
University degree^7^ (%)	10.5	11.8	13.6	15.0	15.3
Energy intake (kcal/d)	2039 (414)	2384 (410)	2616 (460)	2842 (538)	3294 (742)
Bodyweight (kcal/kg)	25.9 (6.8)	30.1 (7.1)	32.8 (7.9)	35.4 (8.8)	40.5 (11.4)
Fat E%^8^	37.3 (6.3)	38.3 (6.1)	39.2 (6.1)	40.0 (6.2)	40.8 (6.1)
Saturated fat E%^8^	15.3 (3.6)	16.1 (3.8)	16.7 (3.9)	17.3 (4.1)	17.8 (4.1)
Unsaturated fat E%^8^	19.5 (3.6)	19.7 (3.3)	20.1 (3.3)	20.3 (3.4)	20.5 (3.2)
Protein E%^8^	14.8 (2.4)	15.0 (2.3)	15.2 (2.3)	15.4 (2.4)	16.0 (2.6)
Carbohydrate E%^8^	48.0 (6.4)	46.7 (6.1)	45.6 (6)	44.6 (6)	43.2 (5.9)
Dietary fiber (g)	18.4 (7.1)	20.3 (6.7)	21.5 (7.2)	22.3 (7.3)	24.4 (8.5)
Dietary fiber (g/1000 kcal)	9.3 (2.9)	8.9 (2.5)	8.6 (2.4)	8.3 (2.3)	7.9 (2.2)

1 Carbon dioxide equivalents.

2 Quintiles of dietary GHGE reported as kg carbon dioxide equivalents (CO_2_eq)/d for females/males 1: <4.1/<5.0, 2: 4.1–4.8/5.0–5.9, 3: 4.8–5.6/5.9–6.9, 4: 5.7–6.5/6.9–8.2, 5: >6.5/>8.2. Values are means (SD) or percentages.

3 BMI, based on 25,929 participants (41 missing).

4 Based on 25,961 (9 missing).

5 Based on 25,949 participants (21 missing). High alcohol consumption defined as >30 g/d for females and >40 g/d for men.

6 Based on 25,838 participants (132 missing). Highest leisure time physical activity quintile.

7 Based on 25,904 (66 missing).

8 Nonalcoholic energy percentage of intake.

**TABLE 2:** Daily consumption of different food groups in grams and their contribution to proportion (%) of greenhouse gas emissions (GHGE) among 25,970 participants from the Malmö Diet and Cancer Study.

	Food intake (g/d) by quintiles of dietary GHGE/d^1^					
	1, *n* (%)	2, *n* (%)	3, *n* (%)	4, *n* (%)	5, *n* (%)	All, *n* (%)
Females (*n*)	3163	3163	3164	3163	3163	15,816
Vegetables	226 (7.3)	256 (6.5)	275 (6.1)	301 (5.9)	340 (5.4)	279 (6.2)
Fruit, berries, nuts	175 (7.4)	198 (6.4)	203 (5.6)	218 (5.2)	227 (4.3)	204 (5.8)
Cereals	107 (5.3)	119 (4.5)	126 (4.1)	135 (3.8)	146 (3.3)	127 (4.2)
Red meat	66 (27)	83 (30)	96 (32)	106 (34)	135 (39)	97 (32)
Poultry and egg	30 (2.9)	34 (2.5)	37 (2.3)	40 (2.1)	44 (1.8)	37 (2.3)
Seafood	34 (6.1)	39 (6.8)	42 (7.4)	45 (7.9)	51 (9.0)	42 (7.4)
Dairy	312 (25)	383 (25)	420 (24)	449 (23)	510 (22)	415 (24)
Added fats	20 (1.6)	22 (1.3)	24 (1.2)	25 (1.1)	26 (1.0)	24 (1.3)
Nonalcoholic drinks	1445 (8)	1509 (8)	1575 (8)	1660 (7)	1756 (7)	1589 (8)
Alcoholic drinks	79 (3.5)	111 (4)	130 (4.1)	153 (4.2)	191 (4.2)	133 (4)
Sweet and snacks	71 (5.3)	81 (4.9)	85 (4.6)	93 (4.4)	102 (4)	86 (4.6)
Other foods	16 (0.6)	21 (0.6)	21 (0.5)	22 (0.5)	25 (0.4)	21 (0.5)
Males (*n*)	2030	2031	2031	2031	2031	10,154
Vegetables	266 (6.3)	299 (5.6)	319 (5.2)	342 (4.8)	395 (4.4)	324 (5.3)
Fruit, berries, nuts	150 (5.1)	168 (4.4)	171 (3.8)	176 (3.3)	185 (2.7)	170 (3.9)
Cereals	154 (6.1)	173 (5.3)	186 (4.9)	197 (4.4)	218 (3.8)	186 (4.9)
Red meat	99 (31)	123 (35)	141 (37)	163 (40)	215 (45)	148 (38)
Poultry and egg	37 (2.8)	40 (2.3)	42 (2.1)	47 (2.0)	52 (1.7)	43 (2.2)
Seafood	43 (5.7)	47 (6.3)	50 (6.3)	51 (6.4)	59 (7.1)	50 (6.4)
Dairy	348 (23)	420 (22)	466 (22)	514 (21)	577 (19)	465 (22)
Added fats	29 (1.7)	33 (1.5)	35 (1.4)	36 (1.3)	37 (1)	34 (1.4)
Nonalcoholic drinks	1282 (7)	1323 (6)	1356 (6)	1442 (6)	1551 (5)	1391 (6)
Alcoholic drinks	179 (5.8)	252 (6.4)	292 (6.4)	325 (6.2)	406 (6)	291 (6.2)
Sweet and snacks	87 (5)	97 (4.5)	103 (4.3)	106 (3.8)	116 (3.3)	102 (4.2)
Other foods	18 (0.5)	21 (0.4)	22 (0.4)	23 (0.3)	26 (0.3)	22 (0.4)

1 Quintiles of dietary GHGE reported as kg carbon dioxide equivalents (CO_2_eq)/d for females/males 1: <10/<5.0, 2: 4.1–4.8/5.0–5.9, 3: 4.8–5.6/5.9–6.9, 4: 5.7–6.5/6.9–8.2, 5: >6.5/>8.2 kg.

Grouping participants according to their GHGE per 1000 kcal, the participants in Q5 were more likely to be younger, have a BMI >25, have a high alcohol consumption, and have a university degree, compared with Q1 (Table 3). Q5 reported a lower energy intake and a lower energy intake per kg body weight and a higher E% of protein. Measured in grams per day, the intake of protein was higher in diets with higher GHGE (Q5/Q1 females; 78 g/73 g, males; 98 g/92 g). The mean GHGE in Q5 was 54% higher for females and 64% for males compared with Q1 (Figure 2B). Consumption of red meat was substantially higher in Q5 compared with Q1 for both females and males (Table 4). However, consumption of fruit, cereals, dairy, added fats, sweets, and snacks were lower in Q5 than Q1.

**TABLE 3:** Participant characteristics according to quintiles of dietary greenhouse gas emissions (GHGE) per 1000 kcal in 25,970 participants from the Malmö Diet and Cancer Study.

	Quintiles of dietary climate impact (kg CO_2_eq^1^/1000 kcal)^2^				
	1	2	3	4	5
Female (*n*)	3163	3163	3164	3163	3163
Age (y)	59.4 (8.0)	58.5 (8.1)	57.7 (8.1)	56.6 (7.8)	55.1 (7.2)
BMI^3^	25.0 (4.3)	25.3 (4.1)	25.5 (4.3)	25.6 (4.2)	25.8 (4.3)
BMI >25^3^ (%)	42.6	45.6	47.2	48.4	51.2
Current smokers^4^ (%)	24.4	27.7	26.8	29.2	32.8
High alcohol consumption^5^ (%)	0.8	1.3	2.2	2.8	4.9
High physical activity^6^ (%)	20.8	20.3	21.0	19.0	20.3
University degree^7^ (%)	11.7	12.9	13.7	17.5	19.4
Energy intake (kcal/d)	2182 (551)	2113 (509)	2022 (459)	1988 (469)	1850 (443)
Bodyweight (kcal/kg)	33.8 (10.4)	32.2 (9.7)	30.4 (8.7)	29.7 (8.6)	27.5 (8.2)
Fat E%^8^	37.8 (6.4)	38.2 (5.8)	37.8 (5.9)	37.8 (6.1)	37.3 (6.1)
Saturated fat E%^8^	16.3 (4.0)	16.8 (3.8)	16.6 (3.8)	16.5 (3.9)	16.0 (3.6)
Unsaturated fat E%^8^	19.1 (3.4)	19.0 (3.0)	18.8 (3.0)	19.0 (3.1)	18.9 (3.2)
Protein E%^8^	13.8 (2.0)	14.9 (1.9)	15.6 (2.0)	16.2 (2.1)	17.7 (2.4)
Carbohydrate E%^8^	48.4 (6.3)	47.0 (5.7)	46.5 (5.8)	45.9 (6.0)	45.0 (6.0)
Dietary fiber (g)	20.5 (7.2)	19.3 (6.3)	18.7 (5.7)	18.4 (5.9)	17.4 (5.5)
Dietary fiber (g/1000 kcal)	9.7 (3.1)	9.5 (2.6)	9.6 (2.6)	9.7 (2.7)	10.0 (2.8)
Male (*n*)	2030	2031	2031	2031	2031
Age (y)	61.4 (7.1)	60.6 (7.1)	59.3 (7.0)	58.5 (6.9)	57.1 (6.6)
BMI^3^	25.7 (3.4)	26.2 (3.4)	26.2 (3.4)	26.5 (3.5)	26.8 (3.6)
BMI >25^3^ (%)	55.0	61.6	62.4	64.2	68.8
Current smokers^4^ (%)	26.0	28.6	28.0	31.2	29.8
High alcohol consumption^5^ (%)	2.4	5.6	7.4	10.3	11.6
High physical activity^6^ (%)	23.6	22.3	20.5	19.3	16.3
University degree^7^ (%)	9.9	11.9	14.5	12.9	16.8
Energy intake (kcal/d)	2808 (726)	2718 (680)	2635 (646)	2576 (637)	2439 (631)
Bodyweight (kcal/kg)	36.1 (10.6)	34.1 (10.0)	32.9 (9.4)	31.8 (9.1)	29.8 (9.0)
Fat E%^8^	38.5 (6.5)	39.4 (6.2)	39.4 (6.3)	39.1 (6.2)	39.1 (6.3)
Saturated fat E%^8^	16.2 (4.0)	16.9 (4.0)	16.9 (4.1)	16.7 (4.0)	16.5 (3.9)
Unsaturated fat E%^8^	20.0 (3.6)	20.0 (3.3)	20.1 (3.3)	20.0 (3.3)	20.1 (3.3)
Protein E%^8^	13.5 (1.9)	14.5 (1.9)	15.2 (2.0)	15.9 (2.2)	17.2 (2.4)
Carbohydrate E%^8^	48.0 (6.3)	46.1 (6.0)	45.4 (6.1)	45.0 (6.0)	43.7 (6.1)
Dietary fiber (g)	24.1 (9.0)	22.0 (7.4)	21.0 (7.2)	20.5 (7.0)	19.2 (6.5)
Dietary fiber (g/1000 kcal)	8.9 (2.8)	8.5 (2.4)	8.5 (2.4)	8.5 (2.5)	8.5 (2.5)

1 Carbon dioxide equivalents.

2 Quintiles of dietary GHGE reported as kg carbon dioxide equivalents (CO_2_eq)/d for females/males 1: <2.2/<2.1, 2: 2.2–2.5/2.1–2.3, 3: 2.5–2.7/2.3–2.6, 4: 2.7–3.1/2.6–3.0, 5: >3.1/>3.0 kg CO_2_eq. Values are means (SD) or percentages.

3 BMI, based on 25,929 participants (41 missing).

4 Based on 25,961 (9 missing).

5 Based on 25,949 participants (21 missing). High alcohol consumption defined as >30 g/d for females and >40 g/d for men.

6 Based on 25,838 participants (132 missing). Highest leisure time physical activity quintile.

7 Based on 25,904 (66 missing).

8 Nonalcoholic energy percentage of intake.

**TABLE 4:** Daily consumption of different food groups in grams and their contribution to proportion (%) of dietary greenhouse gas emissions (GHGE) among 25,970 participants from the Malmö Diet and Cancer Study.

	Food intake (g/d) by quintiles of dietary GHGE/1000 kcal^1^				
	1, *n* (%)	2, *n* (%)	3, *n* (%)	4, *n* (%)	5, *n* (%)
Females (*n*)					
Vegetables	263 (6.9)	270 (6.3)	275 (6.2)	288 (6.1)	301 (5.8)
Fruit, berries, nuts	203 (7.1)	204 (6.1)	206 (5.8)	208 (5.3)	200 (4.5)
Cereals	150 (6)	135 (4.7)	124 (4)	119 (3.5)	105 (2.8)
Red meat	76 (22.6)	91 (28.6)	95 (31.8)	104 (36)	120 (42.5)
Poultry and egg	35 (2.7)	37 (2.5)	38 (2.3)	38 (2.1)	38 (1.9)
Seafood	39 (5.4)	41 (6.5)	43 (7.3)	44 (8.2)	46 (9.9)
Dairy	416 (28.9)	435 (26.5)	429 (24.4)	416 (21.7)	378 (17.2)
Added fats	29 (1.8)	25 (1.4)	22 (1.2)	22 (1.1)	20 (0.9)
Nonalcoholic drinks	1489 (7.6)	1525 (7.8)	1572 (7.8)	1642 (7.6)	1718 (7.1)
Alcoholic drinks	91 (3.3)	119 (3.9)	139 (4.3)	147 (4.2)	168 (4.4)
Sweet and snacks	113 (7)	96 (5.3)	85 (4.4)	77 (3.8)	61 (2.7)
Other foods	23 (0.7)	23 (0.6)	21 (0.5)	20 (0.4)	17 (0.3)
Males (*n*)					
Vegetables	311 (6)	313 (5.4)	321 (5.3)	332 (5.1)	343 (4.6)
Fruit, berries, nuts	175 (5)	173 (4.2)	171 (3.9)	169 (3.5)	162 (2.8)
Cereals	226 (7.2)	196 (5.4)	181 (4.7)	171 (4)	153 (3.2)
Red meat	119 (27.9)	137 (33.3)	145 (37)	157 (41.8)	182 (49.2)
Poultry and egg	43 (2.7)	43 (2.4)	44 (2.2)	44 (2)	43 (1.6)
Seafood	49 (4.9)	49 (5.8)	50 (6.3)	50 (6.6)	52 (8.1)
Dairy	470 (26.2)	493 (24.4)	480 (22.2)	471 (19.6)	411 (15.4)
Added fats	42 (2)	35 (1.5)	34 (1.3)	31 (1.1)	28 (0.9)
Nonalcoholic drinks	1360 (6.3)	1367 (6.2)	1405 (6.1)	1393 (5.9)	1429 (5.1)
Alcoholic drinks	198 (5.1)	268 (6.2)	302 (6.6)	334 (6.7)	353 (6.2)
Sweet and snacks	131 (6.2)	113 (4.8)	100 (4)	91 (3.4)	74 (2.5)
Other foods	24 (0.5)	23 (0.5)	20 (0.4)	21 (0.3)	21 (0.3)

1 Quintiles of dietary GHGE reported as kg carbon dioxide equivalents (CO_2_eq) per 1000 kcal for females/males 1: <2.2/<2.1, 2: 2.2–2.5/2.1–2.3, 3: 2.5–2.7/2.3–2.6, 4: 2.7–3.1/2.6–3.0, 5: >3.1/>3.0 kg CO_2_eq.

Consumption of red meat and seafood (grams per day), as well as their proportion of dietary GHGE, was higher in Q5, both when defining the quintiles based on GHGE per day and per 1000 kcal (TABLE 2, TABLE 4). Consumption of sweets and snacks was higher in absolute amounts but slightly lower as a percentage of total GHGE when defining the quintiles per day. When defining quintiles per 1000 kcal, both the absolute amount and percentage of GHGE from sweets and snacks were higher in Q5.

### Micronutrient intake

#### GHGE per day as exposure

**FIGURE 3:**
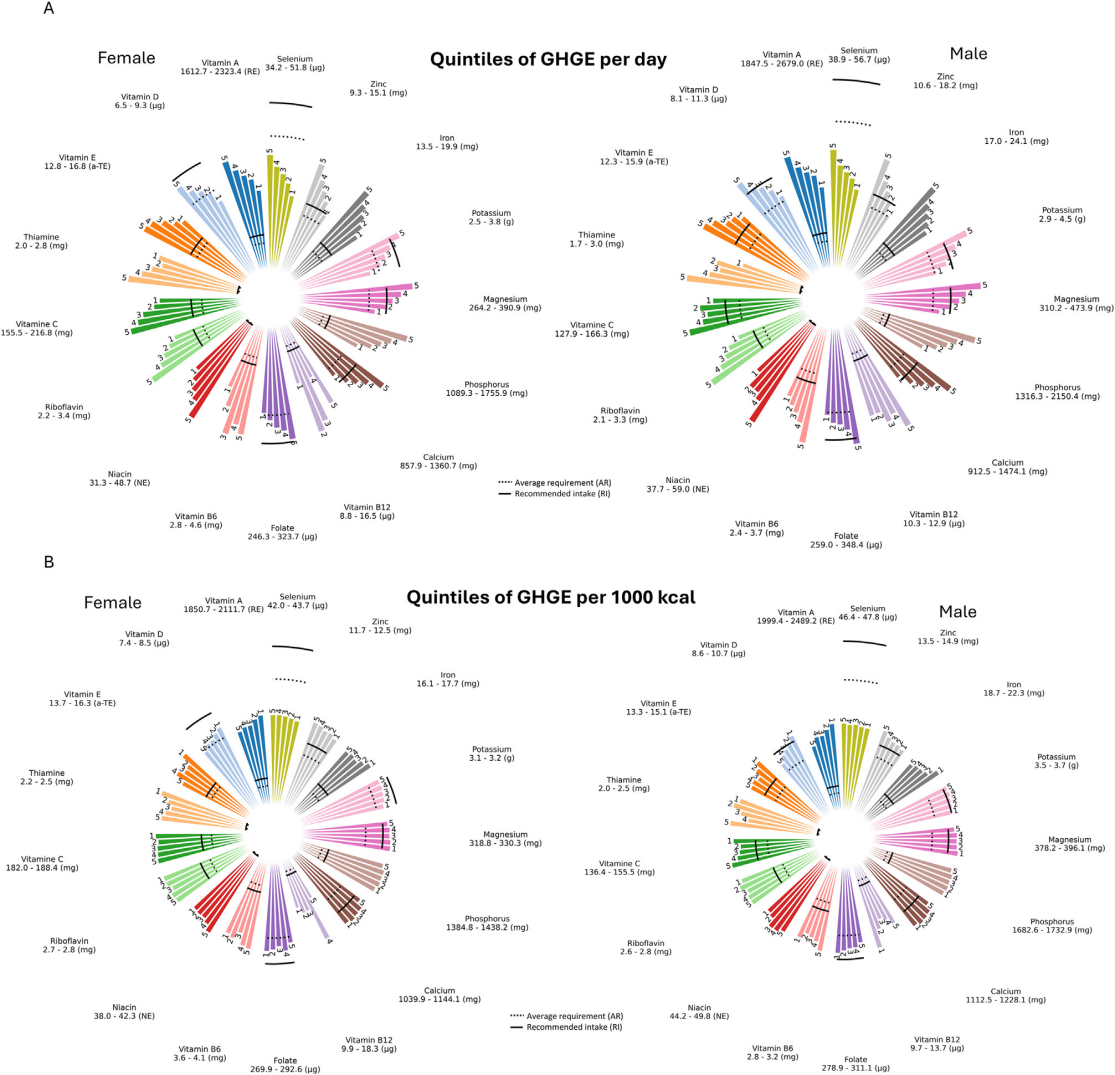
(A) Micronutrient dietary intake using different exposures of greenhouse gas emissions (GHGE),^1^ for 15,816 females in the Malmö Diet and Cancer Study. 1. GHGE reported as kg carbon dioxide equivalents (CO2eq). 2. Analyses are based on linear regression, adjusted for age, season, and dietary assessment version. 3. The different columns report different energy adjustment methods. Significant negative associations are highlighted with red color, and significant positive associations with green color (*P* < 0.05). 4. Retinol equivalents. 5. Alpha-tocopherol equivalents. 6. Niacin equivalents. (B) Micronutrient intake using different exposures of dietary greenhouse gas emissions (GHGE),^1^ reported as kg carbon dioxide equivalents (CO_2_eq), for 10,154 males in the Malmö Diet and Cancer Study. 1. GHGE reported as kg carbon dioxide equivalents (CO2eq). 2. Analyses are based on linear regression, adjusted for age, season, and dietary assessment version. 3. The different columns report different energy adjustment methods. Significant negative associations are highlighted with red color, and significant positive associations with green color (*P* < 0.05). 4. Retinol equivalents. 5. Alpha-tocopherol equivalents. 6. Niacin equivalents.

**FIGURE 4:**
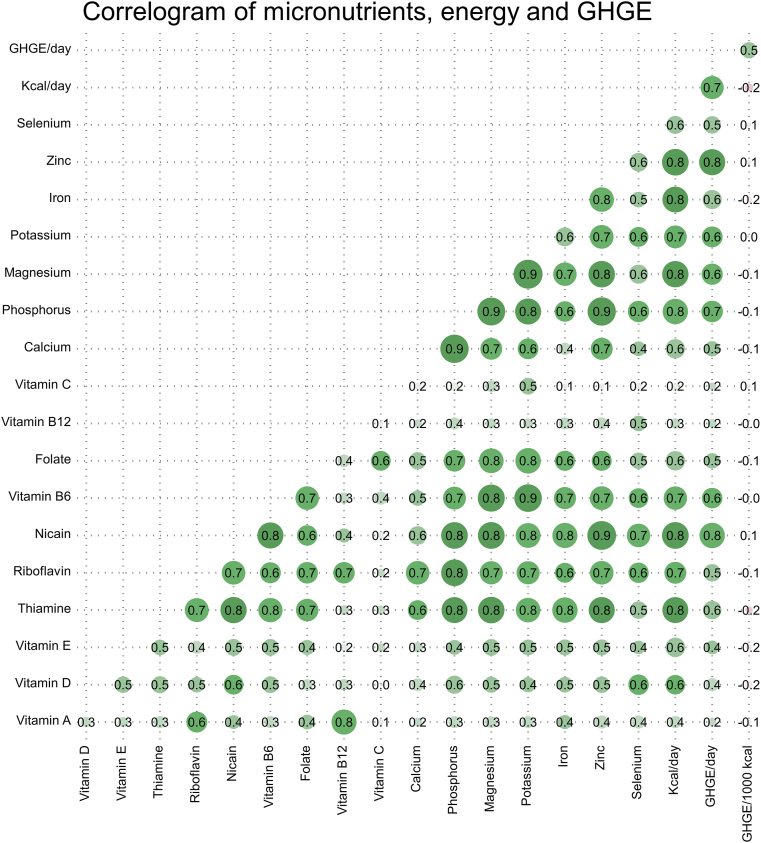
(A) Micronutrient intake per day using quintiles of dietary greenhouse gas emissions (GHGE) per day in the Malmö Diet and Cancer Study. Results are based on linear regression with estimated marginal means, adjusted for age, season, and dietary assessment version. (B) Micronutrient intake per day using quintiles of greenhouse gas emissions (GHGE) per 1000 kcal in the Malmö Diet and Cancer Study. Results are based on linear regression with estimated marginal means, adjusted for age, season, and dietary assessment version.

When comparing the β-values from the multivariate linear regressions, the direction of association between nutrient intake and dietary GHGE depended on whether GHGE per day, GHGE per 1000 kcal, or GHGE per day with different energy adjustments was utilized as the exposure metric (Figure 3A and B). Using GHGE per day as exposure shows positive associations with nutrient intake per day for all micronutrients, indicating that higher GHGE per day is associated with higher intake of these micronutrients. Consequently, a higher proportion of the subjects had a nutrient intake above AR and RI in the higher quintiles of dietary GHGE per day (Figure 4A and Supplemental Figure 3A and B). For both genders, being in Q1 was associated with an intake below the RI for 7 nutrients (vitamin D, folate, calcium, magnesium, potassium, zinc, and selenium), whereas being in Q5 was associated with insufficient intake of 3 nutrients (vitamin D, folate, and selenium) for females and 1 (selenium) for males (Figure 4A).

When the outcome nutrient intake is described as intake per 1000 kcal, the associations show both positive and negative trends. A significant positive association with dietary GHGE per day was found for 4 nutrients in females (niacin, vitamin B_12_, phosphorus, and zinc) and 2 in males (niacin and zinc), whereas negative associations were found for 10 nutrients in both females and males (vitamin A, D, E, thiamine, vitamin B_6_, folate, vitamin C, magnesium, potassium, and iron) as outlined in Figure 3A and B.

#### GHGE per 1000 kcal as exposure

Using GHGE per 1000 kcal as the exposure, without further energy adjustments, mainly showed negative associations with daily micronutrient intake, suggesting that higher GHGE per 1000 kcal is associated with a lower daily intake of these nutrients (Figure 3A and B). However, significant positive associations were seen for niacin, vitamin C, zinc, and selenium, and additionally for vitamin B_12_ and potassium in females. Consequently, having an intake below the RI level was more common in Q5 than Q1 for most micronutrients assessed (Figure 4A and B).

When the exposure is GHGE per 1000 kcal and the outcome is nutrient per 1000 kcal, there was a positive association for all nutrients in females. For males, 13 nutrients showed a positive association and 2 nutrients a negative association.

#### Correlations between energy intakes, macronutrient intakes and GHGE

Positive correlations were observed between total energy intake, and all examined micronutrient intakes. The strongest correlations were observed with thiamine (*r* = 0.80), riboflavin (*r* = 0.74), phosphorus (*r* = 0.79), vitamin B_6_ (*r* = 0.72), magnesium (*r* = 0.82), potassium (*r* = 0.70), iron (*r* = 0.80), and zinc (*r* = 0.83). Weaker correlations were found with vitamin B_12_ (*r* = 0.30) and vitamin C (*r* = 0.18). Similarly, dietary GHGE per day exhibited strong positive correlations with total energy intake (*r* = 0.71), and positive correlations with daily intakes of all examined micronutrient intakes. The strong correlations between energy intake and dietary GHGE per day indicate collinearity between the variables. In contrast, dietary GHGE per 1000 kcal generally showed weak negative correlations with total energy intake and most micronutrients.

#### Adjustments for energy intake using different methods

When including energy as a covariate, the magnitude of the β-values was smaller compared with those from the non-energy adjusted models. The different approaches of energy adjustment were also found to give similar findings irrespective of whether daily nutrient intake or nutrient per 1000 kcal was modeled as the outcome. This contrasts to using daily GHGE or GHGE per 1000 kcal without the inclusion of energy in the model, which resulted in associations in the opposite direction depending on whether the outcome was expressed as daily intake or as intake per 1000 kcal.

The estimates from the standard method of energy adjustment were identical to the estimates from the model with residual energy adjustments and energy as a covariate (Figure 3A and B). In females, these models revealed positive associations with all minerals (calcium, phosphorous, magnesium, potassium, iron, zinc, and selenium) and water-soluble vitamins (thiamine, riboflavin, niacin, vitamin B_6_, folate, vitamin B_12_, and vitamin C), whereas a negative association was observed for vitamin E. There was no significant association for vitamins A and D. In males, there was a positive association with 11 nutrients, but a negative association with the fat-soluble vitamins (vitamins A, D, and E). There was no difference in the direction of the estimates when nutrient intake per 1000 kcal was the outcome. Using the residual model without further energy adjustment in females, there were positive associations for all minerals and water-soluble vitamins except thiamine for both outcomes. The estimates for vitamins A, D, and E were negative when nutrient intake per day was the outcome, and nonsignificant for the micronutrient intake per 1000 kcal. In males, the residual model without energy adjustment showed negative associations for the fat-soluble vitamins and thiamine, and a positive association with 11 nutrients. For iron and folate, the associations were nonsignificant with both outcomes.

For the residual adjustment, GHGE per 1000 kcal adjusted for energy, and daily GHGE adjusted for energy, the results showed an opposite direction compared with GHGE per 1000 kcal without additional energy adjustments. When energy was included as a covariate in the model with GHGE per 1000 kcal as the exposure, the direction of the associations became similar for outcomes measured as daily intake and intake per 1000 kcal. The direction of the estimates also aligned with those from the standard method and the residual method with additional energy adjustments. However, all energy adjustment models produced associations that, for several nutrients, differed substantially from those observed when using dietary GHGE per day.

### Sensitivity analyses

When modeling the GHGE per day and GHGE per 1000 kcal as quintiles, the directions and the magnitude of the associations remained similar to the model using the continuous variable (Supplemental Figure 2A and B).

There was a bigger proportion of misreporters in Q1 compared with all other quintiles when defining the quintiles as dietary GHGE per day (Supplemental Figure 4). On the contrary, more participants were classified as misreporters in Q5 when basing the quintiles on dietary GHGE per 1000 kcal. We observed similar patterns of associations between dietary GHGE and micronutrient intakes when excluding misreporters (Supplemental Figure 3A and B).

## Discussion

### Choice of method influences associations between dietary climate impact and nutrient intake

Different methodologies for modeling and reporting dietary GHGE lead to varying results and conclusions. In our study, there was a strong correlation between energy intake and GHGE per day. Reporting climate impact using GHGE per day was associated with higher daily micronutrient intake. When including energy in the model, the magnitude of the associations became less pronounced, and the result for GHGE per 1000 kcal was similar to those from GHGE per day. Conversely, using GHGE per 1000 kcal without energy in the model revealed that higher emissions were often associated with lower daily micronutrient intake, although results varied for specific minerals and vitamins. The different energy adjustment models attenuated the associations between daily dietary GHGE and micronutrient intakes. This study underscores that whether and how energy intake is accounted for can alter the perceived relationships between dietary GHGE and micronutrient intake.

Conflicting conclusions about diets’ environmental and nutritional implications in the literature may stem from these inconsistencies in GHGE reporting methods. Two systematic reviews highlight this variability. Leonard et al. [5]’s review suggests that lower-GHGE diets tend to result in lower micronutrient intakes, potentially leading to inadequate levels of essential nutrients. Payne et al. [31] have a similar conclusion, which is however mainly based on modeling studies using varied approaches for reporting GHGE. Leonard et al. [5] include both energy and non-energy-adjusted GHGE, adherence to the EAT-Lancet diet, and proportion of animal-sourced protein as exposures from observational studies. Additionally, the review includes a mix of observational studies, modeling studies, and 1 randomized controlled trial (RCT). By integrating findings from our study with observations from previous reviews, it becomes evident that the way GHGE is modeled and reported significantly influences the perceived relationship between dietary GHGE and micronutrient intake. This underscores the importance of transparency in GHGE reporting methods to facilitate more consistent and comparable assessments of the environmental and nutritional impacts of diets, and, in the longer term, potentially establish a standardized approach.

### GHGE per day: estimating total environmental impact

Calculating GHGE per day estimates the total climate impact based on food quantity and type, often correlating positively with micronutrient intake due to higher consumption of food [8]. Although self-reported dietary data may include extreme values from true findings or misreporting, grouping participants into quintiles normalizes these values, which might overcome the problem. Because micronutrient recommendations are based on absolute amounts, assessing nutrient adequacy requires an approach that considers total intake. Dietary GHGE per day offers a straightforward method for evaluating environmental impact in relation to total food emissions, which directly contribute to carbon footprints. However, not all dietary assessment methods are equally suited for this approach. Self-reported dietary information can never accurately capture complete energy intake [32]. For instance, FFQs are more effective for ranking individuals rather than capturing complete dietary intake [33]. A limitation is that the approach of using GHGE per day is largely affected by diet quantity which may obscure differences in GHGE mainly because of variation in energy intake.

### GHGE per 1000 kcal: assessing environmental efficiency

Reporting GHGE per 1000 kcal attempts to illustrate how food choices affect climate impact for the same caloric intake. Previous studies have reported positive associations between GHGE per 1000 kcal and micronutrient intake per 1000 kcal [10,11], which is confirmed in our study. In our study, there was a negative association between energy intake and GHGE per 1000 kcal. By including energy as a covariate, it may more accurately estimate the relationship for the same caloric intake [17,34] (see Figure 3A and B). Results derived from this method allow comparisons across populations with varying energy intakes and might still be understandable to a wider community [18]. However, the method does not account for overconsumption because it does not capture the total dietary GHGE and may therefore be difficult to communicate and include in carbon budget calculations.

### Energy adjustment in statistical models: interpretational challenges

Adjusting for energy intake in statistical models has shown conflicting results for associations with micronutrient intake in other studies. Although some studies found that energy-adjusted dietary GHGE was associated with higher micronutrient intake [13,14], others found the opposite [12] or mixed associations [15]. In our study, applying different methods of energy adjustment yielded varying results, with all models demonstrating weaker associations between dietary GHGE and nutrient intake compared with GHGE per day, alongside mixed or statistically nonsignificant trends. The standard method and the residual method using energy as a covariate revealed identical results, suggesting that the simpler standard method could be used. Energy intake has been emphasized as a primary factor explaining the differences in dietary climate impact among subgroups within the population [25,[35], [36], [37], [38], [39]], and in nutrient intake variations [18]. Energy adjustment aims to eliminate the effect of differential calorie consumption on outcomes, focusing on diet composition and indirectly adjusting for unmeasured variables like metabolic efficiency. Another reason for adding energy as a covariate is to limit the influence of misreporting. However, this approach has interpretational challenges. It can be challenging to interpret in the context of climate change because it may not account for the carbon footprint of overconsumption, making the total dietary environmental impact difficult to assess. Additionally, adjustment for energy intake changes the interpretation of the model estimates such that they represent substitutions between foods with different amounts of GHGE, while maintaining the same energy intake. Deciding to adjust for energy is context-specific, and its relevance for the research question should be considered, in particular whether researchers are interested in capturing the effect of overconsumption or the effect of making food substitutions without altering the overall caloric intake. Finally, in our study, there was a strong correlation between dietary GHGE per day and energy intake (*r* = 0.71) and inclusion of energy as a covariate may therefore introduce errors due to collinearity.

### Assessing nutrient adequacy beyond nutrient intake measurements

Given the challenges in assessing and interpreting micronutrient intake in relation to GHGE, measuring nutrient status may offer a solution. However, this approach is not common, and reliable biomarkers might not exist for all micronutrients. In the systematic review by Leonard et al. [5], only 1 of the 56 included studies had nutrient status as an outcome; the others used nutrient intake. In the study, an RCT assessing micronutrient status, the exposure was based on the percentage of animal-sourced protein and not climate impact per se [40]. A recent study found that despite a lower intake of micronutrients, more climate-friendly diets did not substantially increase the risk of micronutrient deficiencies [9]. Although there was a slight increase in the prevalence of anemia among females, there was no significant difference in the risk of other deficiencies. Additionally, the correlation between nutrient intake and nutrient status was low.

### Recommendations for modeling dietary GHGE and its policy implications

This study contributes to a broader dimension of measuring the health impact of climate-friendly diets to advance the knowledge of nutritional risks and benefits of sustainable diets. To our knowledge, this is the first attempt to systematically compare different methods and examine their implications for results and conclusions on this critical topic. We acknowledge that each method has unique strengths and limitations. Given the novelty of this comparison, we believe further research is essential to better understand the differences, advantages, and limitations of these methods and to develop evidence-based recommendations tailored to specific contexts. Comparing GHGE per day, per 1000 kcal, and energy-adjusted models illuminates distinct insights into environmental and nutritional impacts. However, not all models account for the impact of overconsumption, which may lead to an underestimation of its role in dietary sustainability assessments. On the basis of our results and despite imprecise measures of absolute intakes from self-reports, we therefore suggest that GHGE per day most accurately captures dietary climate impact and its consequences for daily micronutrient intake. Of the energy-adjusted models, we suggest that the standard method captures dietary climate impact in the most accurate and simple way.

Because different methods provide unique insights, employing >1 approach may be necessary to balance dietary environmental sustainability with public health goals and policies. Policymakers and health professionals may need to consider multiple dimensions when promoting sustainable diets. The daily GHGE approach addresses both overconsumption and food choice. Inclusion of energy as a covariate or expressing GHGE per caloric intake highlights the consequences of low-carbon foods in balanced caloric intake. Both perspectives might call for strategies like promoting nutrient-dense, low-emission foods like legumes, whole grains, fruits, vegetables and reducing high-emission, often animal-sourced, foods. Regardless of the modeling approach, it is important to recognize that a positive association between GHGE and nutrient intake does not necessarily imply that higher intake is always better. Even the group with the lowest GHGE may still achieve adequate nutrient intake, meeting or exceeding RI levels. Consequently, higher intake groups should be evaluated in the context of potential overconsumption, which can pose health risks and increase dietary climate impact.

### Strengths and limitations

This study exhibits several strengths, including the utilization of dietary intake data from a large population-based cohort. The dietary data in MDC are based on a rigorous dietary assessment method that combines an FFQ, a food diary, and an interview, providing more detailed insights compared with a conventional FFQ. Additionally, we investigate dietary climate impact as GHGE per day, GHGE per 1000 kcal, and GHGE per day adjusted for energy intake using different methods. We also examine nutrient intake as different outcomes, both per day and per 1000 kcal. To our knowledge, no previous study has elaborated on these different exposures and outcomes using the same data, allowing us to observe how results vary across different approaches. Nevertheless, certain limitations should be mentioned. The dietary data were collected during the 1990s and may not accurately reflect current diets and co-consumption patterns. Fortification and, consequently, the intake of micronutrients might differ when comparing different time periods. In dietary studies, including the MDC, misreporting is a common limitation that may affect the accuracy of estimated dietary GHGE values and nutrient intakes. However, this approach detects misreporting of energy intake rather than specific foods. In this project, it is the reported food intake, and not energy intake per se, which is the basis for calculating both GHGE and micronutrient intake in our study. If participants underreport their food intake, both GHGE and nutrient intake estimates would be proportionally reduced, as both the numerator and denominator are derived from the same reported food data. Therefore, including the potential misreporters of energy provides a more representative analysis of the cohort and helps avoid introducing additional biases from selective corrections for misreporting in either the exposure (GHGE) or the outcome (nutrient intake), which potentially may limit the interpretation and application of findings. This decision of inclusion was supported by the fact that excluding potential energy misreporters in sensitivity analysis did not alter the associations between GHGE and micronutrient intake. The combination of dietary assessment methods used in MDC, while showing high validity (energy-adjusted validity correlation coefficients between 0.28 and 0.77 for micronutrients), makes it more reliable but does not eliminate all sources of measurement uncertainty [23]. The dietary assessment method used in MDC yielded an 18% higher absolute energy intake at the group level compared with the reference method. Although this discrepancy may influence absolute intake comparisons with other cohorts, we believe that it has a small impact on the results of the study, as the relative ranking of participants remains consistent. Although our findings provide valuable insights, they are influenced by the specific characteristics of the dataset and may not fully generalize to other populations or contexts, as the study was conducted in the 1990s with a middle-aged cohort from a high-income country. Future research in more diverse cohorts and settings will be essential for validating these results and refining the modeling approaches.

### Future research

Future research should delve deeper into the complexities of assessing associations between dietary climate impact and nutrient intake, differentiating between various methods of utilizing dietary GHGE as an exposure. Researchers should consider which approach to analyzing GHGE is most appropriate for their context and research questions and be transparent about the methods used. In the existing literature, it is important to distinguish findings related to dietary GHGE or other environmental indicators and proxies for environmental impact for example, the adherence to EAT-Lancet diet, or the balance between animal-sourced and plant-based proteins. Additionally, future studies need to clearly specify whether nutrient adequacy results are derived from self-reported diets or simulation modeling approaches. Furthermore, research should aim to clarify how outcomes from different methodologies should be interpreted and applied in practice. To better answer the question about the nutritional impact of eating climate-friendly diets, more studies assessing nutrient status based on biomarkers, in addition to using nutrient intake, need to be conducted.

### Conclusion

This study demonstrates that whether and how energy intake is accounted for in the modeling process can alter the perceived relationship between GHGE and nutrient intake.

Reporting climate impact as GHGE per day, higher GHGE is associated with higher daily micronutrient intake. When including energy in the model, the associations become less pronounced, with mixed or nonsignificant trends observed for some micronutrients. Given that energy is included in the model, the result for GHGE per 1000 kcal becomes similar to those from GHGE per day. In contrast, using GHGE per 1000 kcal without energy in the model revealed that higher emissions were often associated with lower daily micronutrient intake. Only the method of GHGE per day accounts for the impact of overconsumption, and if the dietary data permit comparison of absolute intakes, this may be the most accurate method to capture dietary climate impact and its implications for micronutrient intake. Of the energy-adjusted models, we suggest that the standard method most accurately and simply captures dietary climate impact based on food choices. However, as each method provides unique insights, the choice of how to model GHGE should be guided by the specific research question, intended interpretation, and a commitment to greater transparency. In some cases, a combination of approaches may be necessary to comprehensively understand dietary sustainability.

## Author contributions

The authors’ responsibilities were as follows – AS, ES, EH, UE: designed the research; AS: conducted the analyses, made the conceptualization, visualization, and writing of the original draft of the manuscript; EH, GT, YB, SJ, ES, UE: contributed to the interpretation of results and revision of the manuscript; UE: primary responsibility for final content; and all authors: read and approved the final manuscript.

## Data availability

Data described in the manuscript can be made available on request pending application and approval by the chair of the steering committee for the cohort.

## Funding

The Swedish Heart-Lung Foundation (nr 20200482), Crafoord Foundation (nr 20210674), Agenda 2030 Graduate School, Lund University. The funding agencies had no influence on the design, analysis, or writing of this paper.

## Conflict of interest

The authors report no conflicts of interest.
