# Supplementary material for: Greenhouse gas emissions in relation to micronutrient intake and implications of energy intake: a comparative analysis of different modeling approaches
Source: Am J Clin Nutr. 2025 Mar 10;121(5):1063–76. doi: 10.1016/j.ajcnut.2025.02.031 (PMC12107493; doi:10.1016/j.ajcnut.2025.02.031)
Supplement: Multimedia component 1 [file mmc1.pdf]

Online supplementary material

Supplement to: Stubbendorff A, et al. Greenhouse gas emissions in relation to micronutrient intake and implications of energy intake: A comparative analysis of different modelling approaches

Supplemental figure 1. Flow chart of participants from the Malmö Diet and Cancer Study.

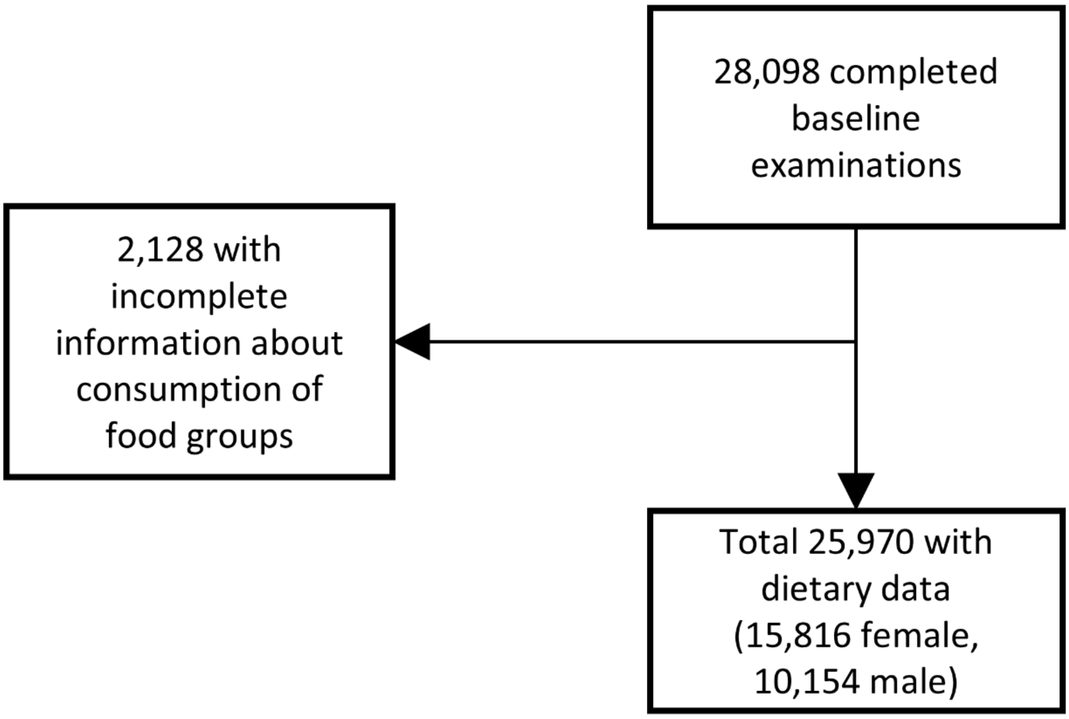

Supplemental figure 2a. Graphs of associations between dietary greenhouse gas emissions (GHGE) and nutrient intake above recommended intake for 15,816 females (12,668 excl. potential mis-reporters) in the Malmö Diet and Cancer Study. Plots are based on estimated marginal means. Coefficients and p-values based on linear regression, adjusted for age, season and dietary assessment version. In one exposure-model energy was added as covariate.

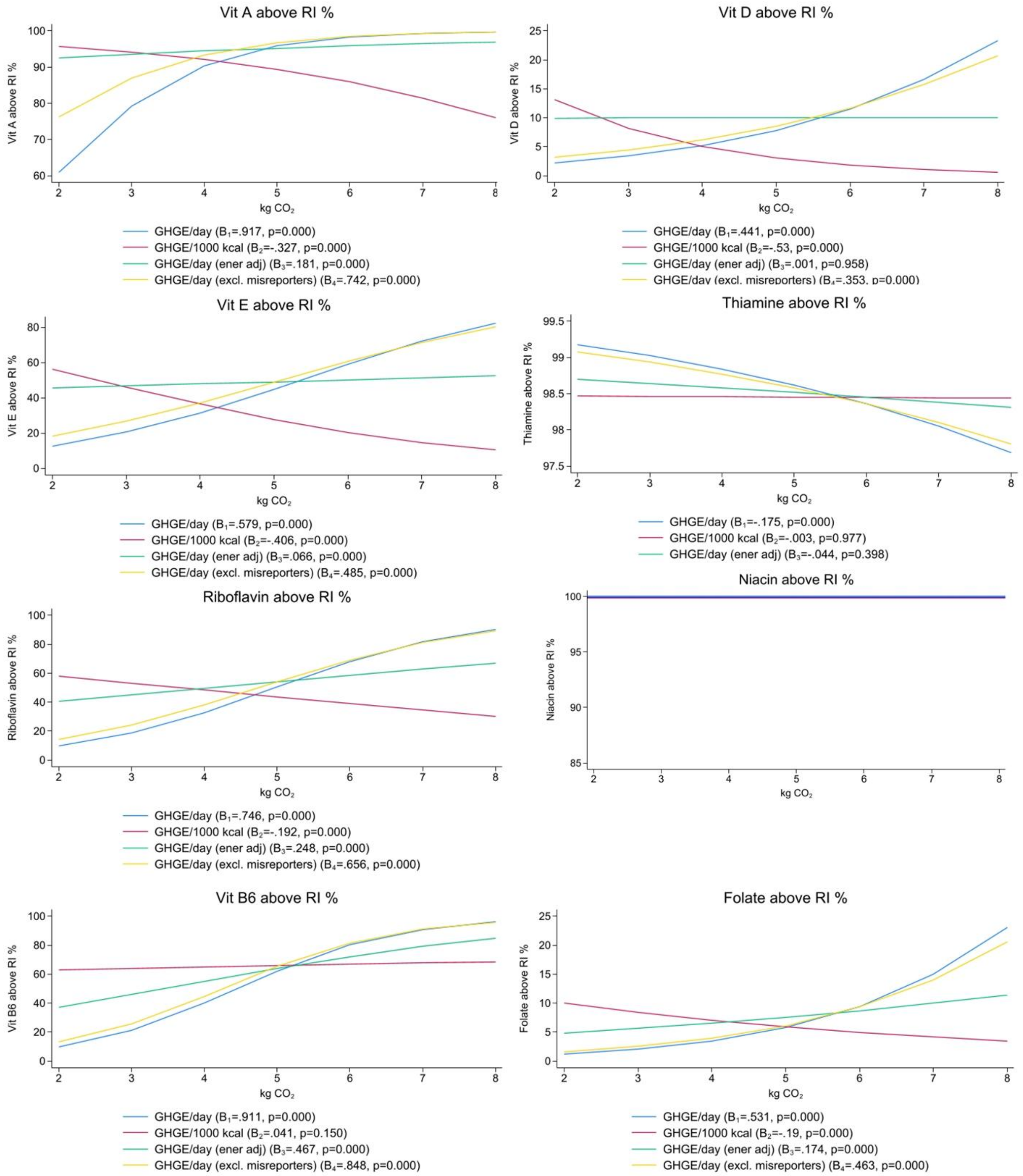

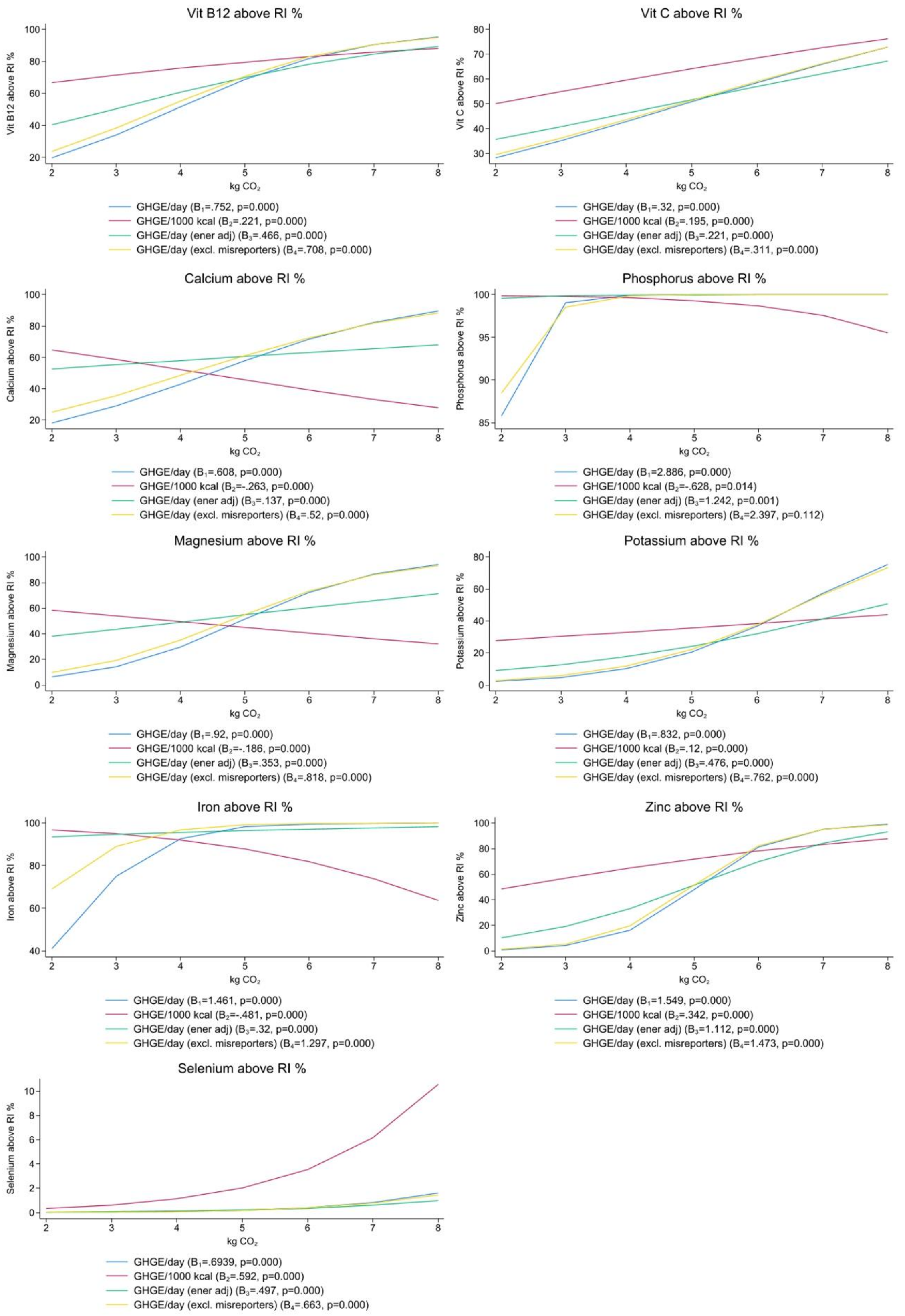
Vit B12 above RI %
Vit B12 above RI %
kg CO_2
GHGE/day (B_1=.752, p=0.000)
GHGE/1000 kcal (B_2=.221, p=0.000)
GHGE/day (ener adj) (B_3=.466, p=0.000)
GHGE/day (excl. misreporters) (B_4=.708, p=0.000)
Vit C above RI %
Vit C above RI %
kg CO_2
GHGE/day (B_1=.32, p=0.000)
GHGE/1000 kcal (B_2=.195, p=0.000)
GHGE/day (ener adj) (B_3=.221, p=0.000)
GHGE/day (excl. misreporters) (B_4=.311, p=0.000)
Calcium above RI %
Calcium above RI %
kg CO_2
GHGE/day (B_1=.608, p=0.000)
GHGE/1000 kcal (B_2=-.263, p=0.000)
GHGE/day (ener adj) (B_3=.137, p=0.000)
GHGE/day (excl. misreporters) (B_4=.52, p=0.000)
Phosphorus above RI %
Phosphorus above RI %
kg CO_2
GHGE/day (B_1=2.886, p=0.000)
GHGE/1000 kcal (B_2=-.628, p=0.014)
GHGE/day (ener adj) (B_3=1.242, p=0.001)
GHGE/day (excl. misreporters) (B_4=2.397, p=0.112)
Magnesium above RI %
Magnesium above RI %
kg CO_2
GHGE/day (B_1=.92, p=0.000)
GHGE/1000 kcal (B_2=-.186, p=0.000)
GHGE/day (ener adj) (B_3=.353, p=0.000)
GHGE/day (excl. misreporters) (B_4=.818, p=0.000)
Potassium above RI %
Potassium above RI %
kg CO_2
GHGE/day (B_1=.832, p=0.000)
GHGE/1000 kcal (B_2=.12, p=0.000)
GHGE/day (ener adj) (B_3=.476, p=0.000)
GHGE/day (excl. misreporters) (B_4=.762, p=0.000)
Iron above RI %
Iron above RI %
kg CO_2
GHGE/day (B_1=1.461, p=0.000)
GHGE/1000 kcal (B_2=-.481, p=0.000)
GHGE/day (ener adj) (B_3=.32, p=0.000)
GHGE/day (excl. misreporters) (B_4=1.297, p=0.000)
Zinc above RI %
Zinc above RI %
kg CO_2
GHGE/day (B_1=1.549, p=0.000)
GHGE/1000 kcal (B_2=.342, p=0.000)
GHGE/day (ener adj) (B_3=1.112, p=0.000)
GHGE/day (excl. misreporters) (B_4=1.473, p=0.000)
Selenium above RI %
Selenium above RI %
kg CO_2
GHGE/day (B_1=.6939, p=0.000)
GHGE/1000 kcal (B_2=.592, p=0.000)
GHGE/day (ener adj) (B_3=.497, p=0.000)
GHGE/day (excl. misreporters) (B_4=.663, p=0.000)

Supplemental figure 2b. Graphs of associations between dietary greenhouse gas emissions (GHGE) and nutrient intake below recommended intake for 10,154 males (6,638 excl potential mis-reporters) in the Malmö Diet and Cancer Study. Plots are based on estimated marginal means. Coefficients and p-values based on linear regression, adjusted for age, season and dietary assessment version. In one exposure-model energy was added as covariate.

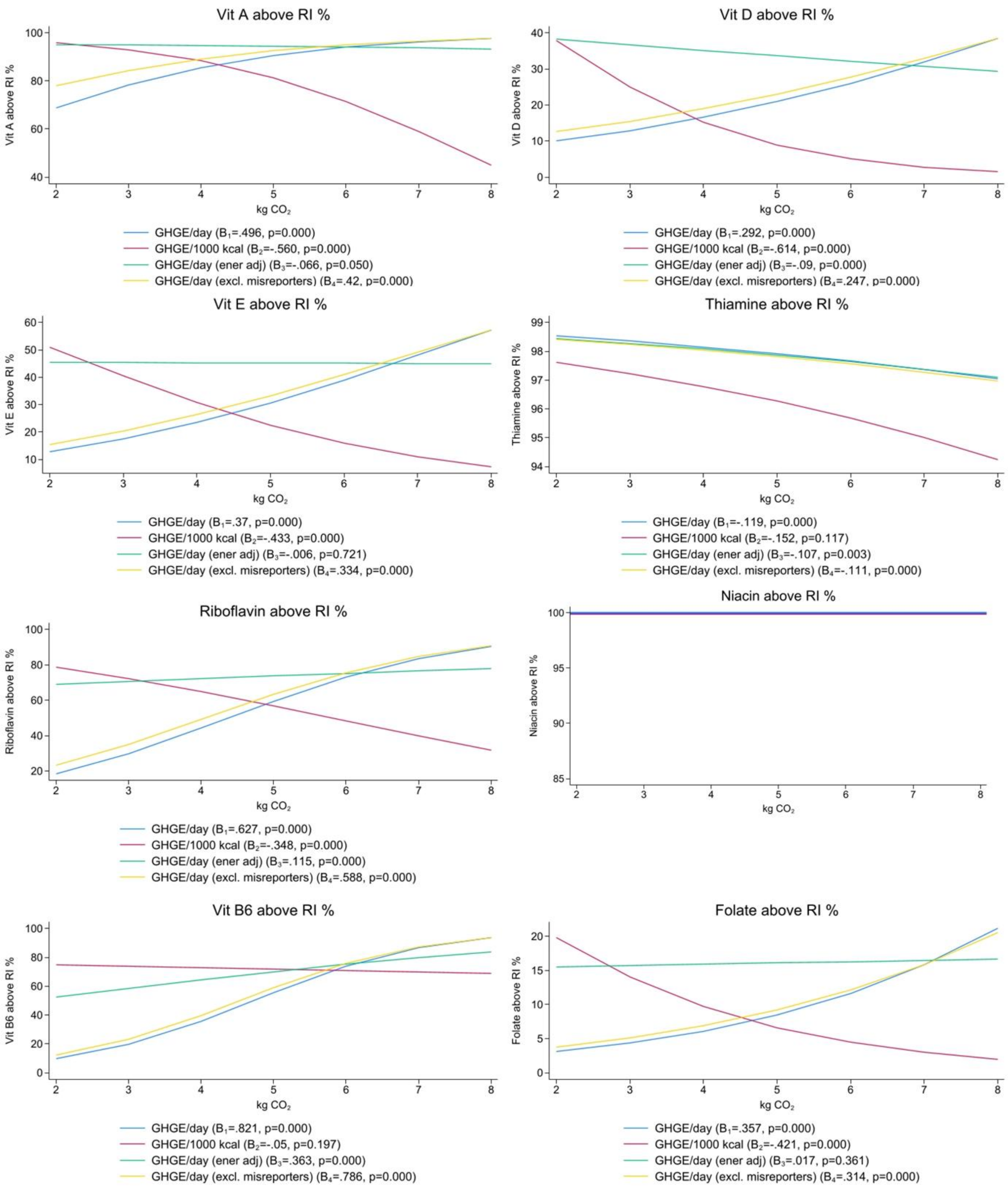

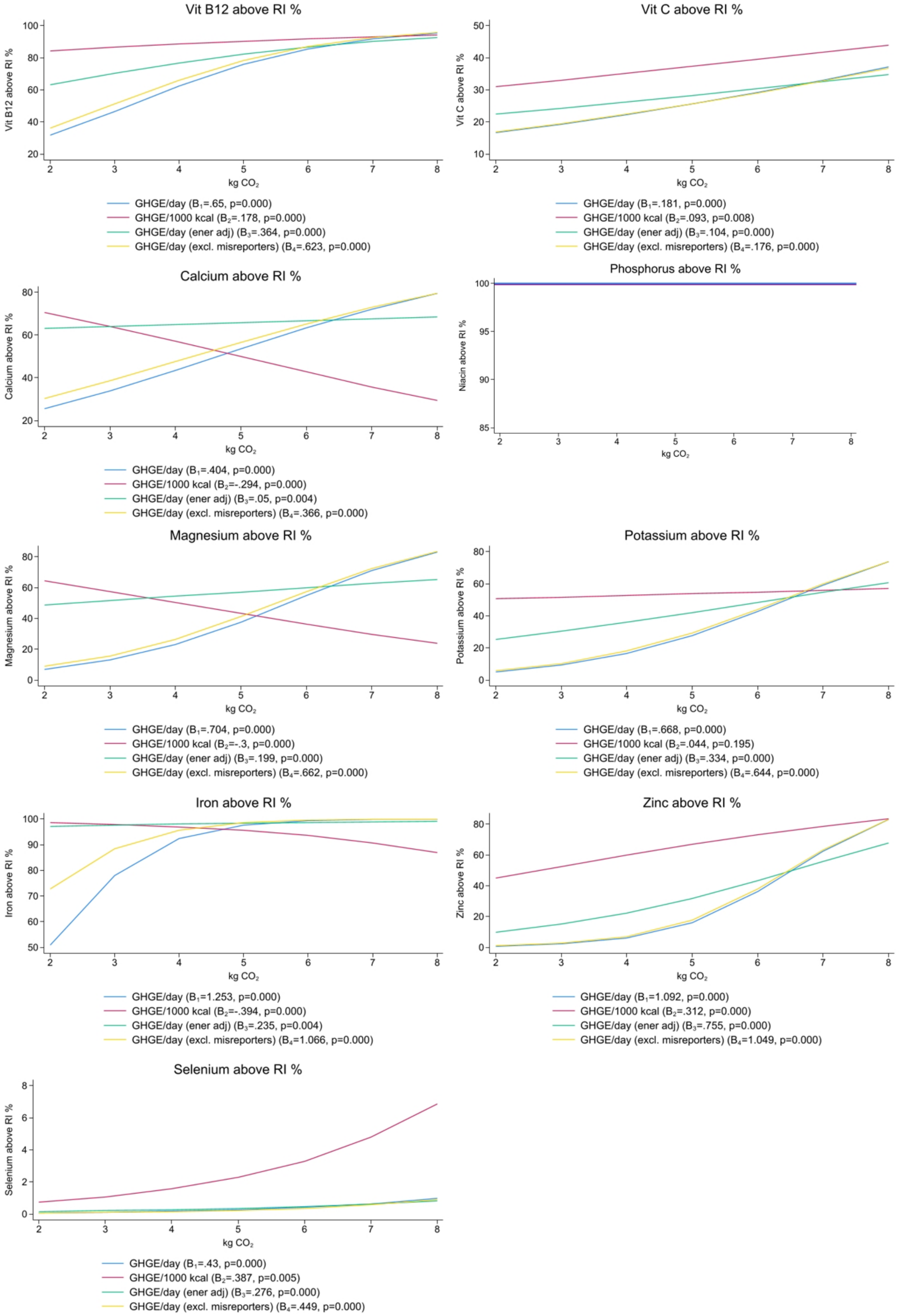
Vit B12 above RI %
Vit B12 above RI %
kg CO_2
GHGE/day (B_1=.65, p=0.000)
GHGE/1000 kcal (B_2=.178, p=0.000)
GHGE/day (ener adj) (B_3=.364, p=0.000)
GHGE/day (excl. misreporters) (B_4=.623, p=0.000)
Vit C above RI %
Vit C above RI %
kg CO_2
GHGE/day (B_1=.181, p=0.000)
GHGE/1000 kcal (B_2=.093, p=0.008)
GHGE/day (ener adj) (B_3=.104, p=0.000)
GHGE/day (excl. misreporters) (B_4=.176, p=0.000)
Calcium above RI %
Calcium above RI %
kg CO_2
GHGE/day (B_1=.404, p=0.000)
GHGE/1000 kcal (B_2=-.294, p=0.000)
GHGE/day (ener adj) (B_3=.05, p=0.004)
GHGE/day (excl. misreporters) (B_4=.366, p=0.000)
Phosphorus above RI %
Niacin above RI %
kg CO_2
Magnesium above RI %
Magnesium above RI %
kg CO_2
GHGE/day (B_1=.704, p=0.000)
GHGE/1000 kcal (B_2=-.3, p=0.000)
GHGE/day (ener adj) (B_3=.199, p=0.000)
GHGE/day (excl. misreporters) (B_4=.662, p=0.000)
Potassium above RI %
Potassium above RI %
kg CO_2
GHGE/day (B_1=.668, p=0.000)
GHGE/1000 kcal (B_2=.044, p=0.195)
GHGE/day (ener adj) (B_3=.334, p=0.000)
GHGE/day (excl. misreporters) (B_4=.644, p=0.000)
Iron above RI %
Iron above RI %
kg CO_2
GHGE/day (B_1=1.253, p=0.000)
GHGE/1000 kcal (B_2=-.394, p=0.000)
GHGE/day (ener adj) (B_3=.235, p=0.004)
GHGE/day (excl. misreporters) (B_4=1.066, p=0.000)
Zinc above RI %
Zinc above RI %
kg CO_2
GHGE/day (B_1=1.092, p=0.000)
GHGE/1000 kcal (B_2=.312, p=0.000)
GHGE/day (ener adj) (B_3=.755, p=0.000)
GHGE/day (excl. misreporters) (B_4=1.049, p=0.000)
Selenium above RI %
Selenium above RI %
kg CO_2
GHGE/day (B_1=.43, p=0.000)
GHGE/1000 kcal (B_2=.387, p=0.005)
GHGE/day (ener adj) (B_3=.276, p=0.000)
GHGE/day (excl. misreporters) (B_4=.449, p=0.000)

Supplemental figure 3a. Micronutrient intake using different exposures of dietary greenhouse gas emissions (GHGE) for 15,816 females in the Malmö Diet and Cancer Study.

	Coefficients for linear regression on different exposures (females)[2,3]	
	Quintiles of GHGE/day[4]	Quintiles of GHGE/1000 kcal[5]
**Micronutrient intake**	**β**	**β**
Vitamin A (RE[6])/day	166 (154,178)	-62.4 (-74.8,-50)
intake/1000 kcal	-12.1 (-18,-6.1)	8.1 (2.2,14)
Vitamin D (µg)	0.7 (0.6,0.7)	-0.2 (-0.3,-0.2)
intake/1000 kcal	-0.03 (-0.04,-0.01)	0.02 (0.01,0.03)
Vitamin E (a-TE[7])	0.9 (0.9,0.9)	-0.4 (-0.4,-0.3)
intake/1000 kcal	-0.05 (-0.06,-0.04)	0.03 (0.02,0.04)
Thiamine (mg)	0.1 (0.1,0.1)	-0.04 (-0.04,-0.04)
intake/1000 kcal	-0.01 (-0.01,-0.01)	0.01 (0.01,0.01)
Riboflavin (mg)	0.2 (0.2,0.2)	-0.02 (-0.03,-0.02)
intake/1000 kcal	0 (0,0)	0.03 (0.02,0.03)
Niacin (NE[8])	3.7 (3.6,3.7)	0.5 (0.4,0.6)
intake/1000 kcal	0.1 (0,0.1)	0.9 (0.9,1)
Vitamin B6 (mg)	0.2 (0.2,0.2)	0 (0,0.01)
intake/1000 kcal	0 (0,0)	0.04 (0.04,0.04)
Folate (µg)	22.4 (21.6,23.1)	-1.9 (-2.8,-1.1)
intake/1000 kcal	-1.5 (-1.9,-1.2)	4.1 (3.7,4.4)
Vitamin B12 (µg)	0.7 (0.7,0.8)	0.1 (0,0.1)
intake/1000 kcal	0.03 (0.01,0.06)	0.2 (0.1,0.2)
Vitamin C (mg)	11.6 (11,12.3)	2.8 (2.2,3.5)
intake/1000 kcal	-0.2 (-0.5,0.1)	3.9 (3.6,4.2)
Calcium (mg)	121 (117,125)	-21.7 (-25.9,-17.5)
intake/1000 kcal	0 (-1.7,1.7)	12.9 (11.3,14.6)
Phosphorus (mg)	161 (158,165)	-8.4 (-12.7,-4.2)
intake/1000 kcal	2.7 (1.2,4.1)	26.4 (25.1,27.8)
Magnesium (mg)	30.2 (29.5,30.8)	-3.7 (-4.5,-2.9)
intake/1000 kcal	-2.4 (-2.7,-2.1)	5.1 (4.8,5.3)
Potassium (g)	0.3 (0.3,0.3)	0.03 (0.02,0.04)
intake/1000 kcal	0 (0,0)	0.1 (0.1,0.1)
Iron (mg)	1.4 (1.4,1.4)	-0.4 (-0.5,-0.4)
intake/1000 kcal	0 (-0.1,0)	0.1 (0.1,0.1)
Zinc (mg)	1.3 (1.3,1.3)	0.2 (0.2,0.2)
intake/1000 kcal	0.1 (0.1,0.1)	0.3 (0.3,0.3)
Selenium (µg)	4 (3.9,4.1)	0.9 (0.8,1)
intake/1000 kcal	0.1 (0,0.2)	1.2 (1.1,1.3)

1. GHGE reported as kg carbon dioxide equivalents (CO2eq). 2. Analyses are based on linear regression, adjusted for age, season and dietary assessment version. 3. The different columns report different energy adjustment methods. Significant negative associations are highlighted with red color, and significant positive associations with green color ($p<0.05$). 4. Quintiles of kg dietary GHGE per day for females 1: <4.1, 2: 4.1-4.8, 3: 4.8-5.6, 4: 5.7-6.5, 5: >6.5. 5. Quintiles of GHGE reported as kg carbon dioxide equivalents (CO_2eq) per 1000 kcal for females 1: <2.2, 2: 2.2-2.5, 3: 2.5-2.7, 4: 2.7-3.1, 5: >3.1 kg CO_2eq. 6. Retinol-equivalents. 7. Alpha-tocopherol equivalents. 8. Niacin equivalents.

Supplemental figure 3b. Micronutrient intake using different exposures of dietary greenhouse gas emissions (GHGE) for 10,154 males in the Malmö Diet and Cancer Study.

	Coefficients for linear regression on different exposures (females)[2,3]	
	Quintiles of GHGE/day[4]	Quintiles of GHGE/1000 kcal[5]
**Micronutrient intake**	**β**	**β**
Vitamin A (RE[6])/day	202 (180,223)	-117.6 (-138.9,-96.4)
intake/1000 kcal	-15.7 (-23.5,-7.8)	-10.8 (-18.5,-3.1)
Vitamin D (µg)	0.9 (0.8,0.9)	-0.5 (-0.5,-0.4)
intake/1000 kcal	-0.1 (-0.1,0)	0 (-0.1,0)
Vitamin E (a-TE[7])	1.1 (1,1.1)	-0.5 (-0.6,-0.5)
intake/1000 kcal	-0.1 (-0.1,-0.1)	0 (-0.02,0.02)
Thiamine (mg)	0.1 (0.1,0.2)	-0.1 (-0.1,-0.1)
intake/1000 kcal	0 (0,0)	0 (0,0)
Riboflavin (mg)	0.2 (0.2,0.2)	0 (-0.1,0)
intake/1000 kcal	0 (0,0)	0 (0,0)
Niacin (NE[8])	4.9 (4.8,5)	0.4 (0.3,0.6)
intake/1000 kcal	0.1 (0.1,0.1)	0.8 (0.8,0.8)
Vitamin B6 (mg)	0.2 (0.2,0.2)	-0.01 (-0.01,0)
intake/1000 kcal	0 (0,0)	0.03 (0.03,0.03)
Folate (µg)	23 (21.9,24.1)	-7.2 (-8.3,-6)
intake/1000 kcal	-2.4 (-2.8,-2)	1.4 (1,1.7)
Vitamin B12 (µg)	1 (0.9,1)	-0.1 (-0.2,0)
intake/1000 kcal	0 (0,0.1)	0.1 (0.1,0.1)
Vitamin C (mg)	8.7 (7.9,9.5)	1.2 (0.3,2)
intake/1000 kcal	-0.9 (-1.2,-0.6)	2 (1.7,2.3)
Calcium (mg)	137 (131,144)	-26.2 (-32.7,-19.8)
intake/1000 kcal	0.5 (-1.5,2.5)	8.6 (6.6,10.5)
Phosphorus (mg)	198 (193,204)	-15.1 (-21.9,-8.4)
intake/1000 kcal	1.5 (-0.3,3.2)	21 (19.3,22.7)
Magnesium (mg)	37.6 (36.5,38.7)	-6.8 (-8.1,-5.5)
intake/1000 kcal	-2.3 (-2.6,-2)	3.4 (3.1,3.7)
Potassium (g)	0.4 (0.4,0.4)	0.01 (0,0.02)
intake/1000 kcal	0 (0,0)	0.1 (0.1,0.1)
Iron (mg)	1.9 (1.8,2)	-0.8 (-0.9,-0.7)
intake/1000 kcal	-0.1 (-0.1,0)	-0.01 (-0.03,0.01)
Zinc (mg)	1.8 (1.8,1.8)	0.3 (0.2,0.3)
intake/1000 kcal	0.1 (0.1,0.1)	0.3 (0.3,0.3)
Selenium (µg)	4.7 (4.5,4.9)	0.5 (0.3,0.7)
intake/1000 kcal	0 (-0.1,0)	0.9 (0.8,0.9)

1. GHGE reported as kg carbon dioxide equivalents (CO2eq). 2. Analyses are based on linear regression, adjusted for age, season and dietary assessment version. 3. The different columns report different energy adjustment methods. Significant negative associations are highlighted with red color, and significant positive associations with green color ($p<0.05$). 4. Quintiles of kg dietary GHGE per day for males 1:<5.0, 2: 5.0-5.9, 3: 5.9-6.9, 4: 6.9-8.2, 5: >8.2 kg. 5. Quintiles of GHGE reported as kg carbon dioxide equivalents (CO_2eq) per 1000 kcal for males 1: <2.1, 2: 2.1-2.3, 3: 2.3-2.6, 4: 2.6-3.0, 5: >3.0 kg CO_2eq. 6. Retinol-equivalents. 7. Alpha-tocopherol equivalents. 8. Niacin equivalents.

Supplemental figure 4. Potential mis-reporters of energy intake in females and males by quintiles defined by dietary greenhouse gas emissions (GHGE) per day and by dietary GHGE per 1000 kcal. 3148 females and 1516 were considered potential mis-reporters.

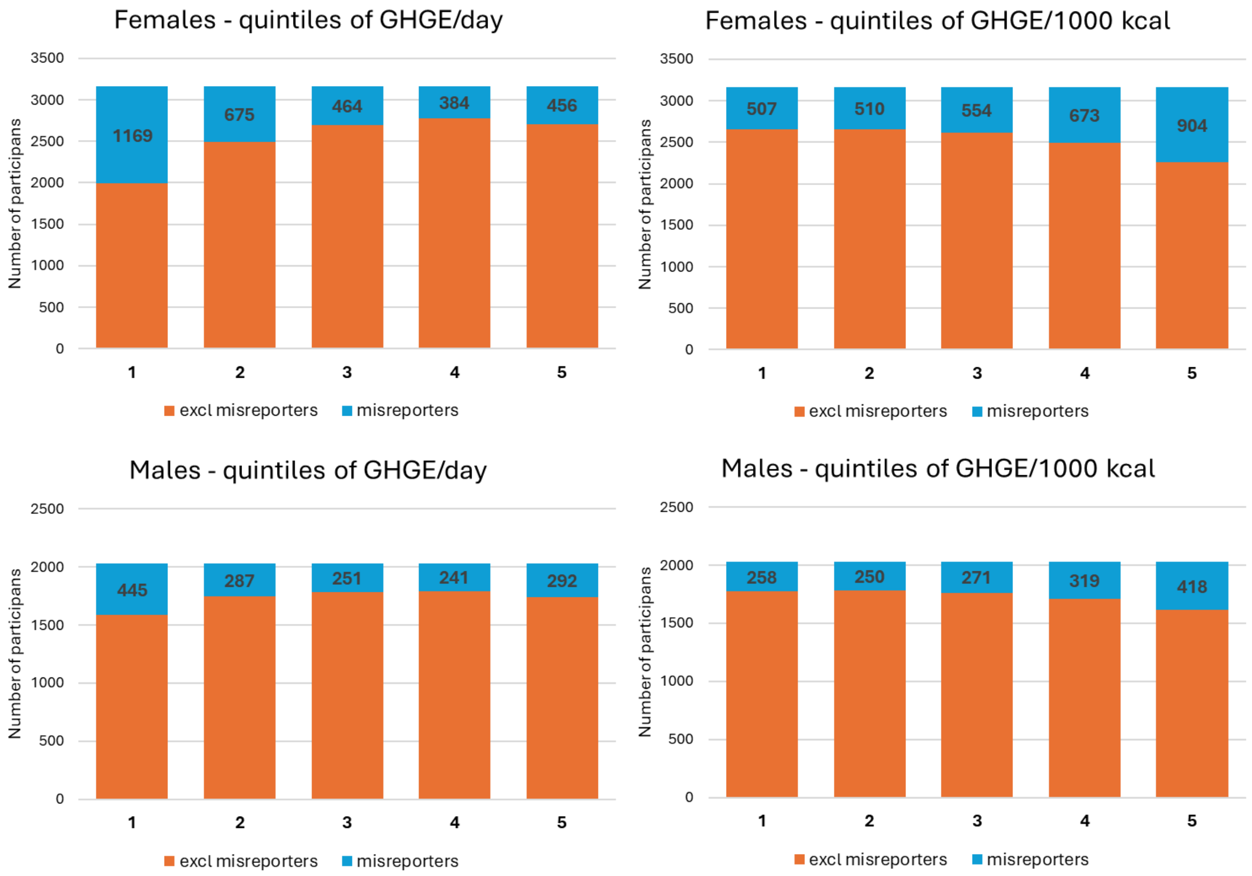

Supplemental table 1. Content of different food groups used in the calculations of dietary greenhouse gas emissions (GHGE) in the Malmö Diet and Cancer Study.

Superior food groups	Food groups	Included foods
Plant-based foods	Vegetables	Vegetables, root vegetables, potatoes, legumes, soy, vegetable juice
	Fruit and berries	Fruit and berries (excluding juices), nuts[1]
	Cereals	Cereals, bread, rice, pasta
	Added fats	Oils, margarines, mayonnaise, dressings
	Other foods	Ketchup, spices, stock, other foods
Animal-sourced foods	Red meat	Beef, pork, lamb, game, processed red meat
	Poultry and egg	Poultry and egg
	Seafood	Fish and shellfish
	Dairy	Milk, fermented milk, cream, cheese, butter, milk-based margarines
Discretionary foods	Non-alcoholic drinks	Coffee, tea, soda, fruit juices, water
	Alcoholic drinks	Beer, wine, spiritus
	Sweet and snacks	Sugar, candy, crisps, jam, cakes, ice cream, sorbet

1. Nuts were included in the fruit and berries group since the mean consumption was very low in this cohort (1.81 grams per day),
